# Cellulose Elementary Fibrils as Deagglomerated Binder for High-Mass-Loading Lithium Battery Electrodes

**DOI:** 10.1007/s40820-024-01642-8

**Published:** 2025-01-21

**Authors:** Young-Kuk Hong, Jung-Hui Kim, Nag-Young Kim, Kyeong-Seok Oh, Hong-I Kim, Seokhyeon Ryu, Yumi Ko, Ji-Young Kim, Kwon-Hyung Lee, Sang-Young Lee

**Affiliations:** 1 Department of Chemical and Biomolecular Engineering, Yonsei University, 50 Yonsei-Ro, Seodaemun-Gu, Seoul, 03722 Republic of Korea; 2 Advanced Analysis and Data Center, Korea Institute of Science and Technology (KIST), Seoul, 02792 Republic of Korea; 3 Ulsan Advanced Energy Technology R&D Center, Korea Institute of Energy Research (KIER), Ulsan, 44776 Republic of Korea; 4 Department of Battery Engineering, Yonsei University, 50 Yonsei-Ro, Seodaemun-Gu, Seoul, 03722 Republic of Korea

**Keywords:** Cellulose elementary fibrils, Deagglomeration, Electrode binders, Lithium batteries, High-mass-loading

## Abstract

Amidst the ever-growing interest in high-mass-loading Li battery electrodes, a persistent challenge has been the insufficient continuity of their ion/electron conduction pathways. Here, we propose cellulose elementary fibrils (CEFs) as a class of deagglomerated binder for high-mass-loading electrodes. Derived from natural wood, CEF represents the most fundamental unit of cellulose with nanoscale diameter. The preparation of the CEFs involves the modulation of intermolecular hydrogen bonding by the treatment with a proton acceptor and a hydrotropic agent. This elementary deagglomeration of the cellulose fibers increases surface area and anionic charge density, thus promoting uniform dispersion with carbon conductive additives and suppressing interfacial side reactions at electrodes. Consequently, a homogeneous redox reaction is achieved throughout the electrodes. The resulting CEF-based cathode (overlithiated layered oxide (OLO) is chosen as a benchmark electrode active material) exhibits a high areal-mass-loading (50 mg cm^–2^, equivalent to an areal capacity of 12.5 mAh cm^–2^) and a high specific energy density (445.4 Wh kg^–1^) of a cell, which far exceeds those of previously reported OLO cathodes. This study highlights the viability of the deagglomerated binder in enabling sustainable high-mass-loading electrodes that are difficult to achieve with conventional synthetic polymer binders.

**Supplementary Information:**

The online version contains supplementary material available at 10.1007/s40820-024-01642-8.

## Highlights

- Cellulose elementary fibrils (CEFs), the most fundamental unit of cellulose, are proposed as a deagglomerated binder for high-mass-loading Li battery electrodes.
- The CEFs, due to their increased surface area and anionic charge density, promote uniform dispersion with carbon additives and mitigate interfacial side reactions in electrodes.
- The CEF-based overlithiated layered oxide cathode exhibits a high areal-mass-loading (50 mg cm^–2^) and a high specific energy density (445.4 Wh kg^–1^) of a cell.

**Supplementary Information:**

The online version contains supplementary material available at 10.1007/s40820-024-01642-8.

## Graphical abstract

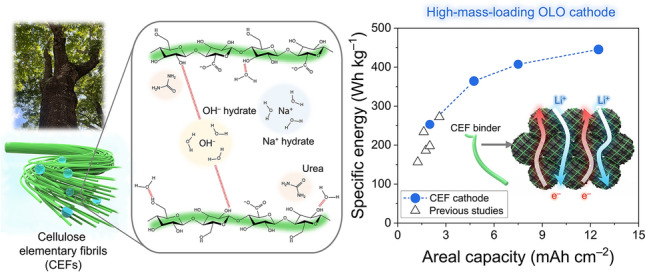

## Introduction

The ongoing surge in demand for smart portable electronics, electric vehicles, and grid-scale energy storage systems has catalyzed the relentless pursuit of high-energy–density lithium (Li) batteries with electrochemical sustainability [1–3]. Many previous studies implemented to reach this goal have concentrated on synthesizing and engineering new electrode active materials [4, 5]. Along with these material-driven approaches, the design of high-mass-loading electrodes has recently emerged as a practical strategy owing to its simplicity and scalability in realizing high-energy–density cells [6–8].

However, a longstanding challenge with the high-mass-loading electrodes has been the difficulty in achieving sufficient interconnectivity of their ion/electron conduction pathways [9–12]. The random and nonuniform intermolecular interactions between electrode components, including carbon conductive additives and polymer binders, often lead to their poor dispersion in electrode slurries [13–17]. This issue becomes more pronounced with the incorporation of carbon nanotubes (CNTs), which tend to aggregate due to their strong van der Waals interactions, thus limiting their effectiveness in forming conductive networks [18]. To address these challenges, both physical methods (e.g., high-shear mixing, three-roll milling) and chemical methods (e.g., surface functionalization, polymer grafting) have been explored [19, 20]. However, these approaches often cause structural damage to the components, require additional dispersants, and complicate fabrication processes, thereby hindering their practical application [21–23]. Consequently, this issue hinders the formation of bi-continuous ion/electron conduction networks across the electrode thickness, resulting in a loss of electrochemical performance and energy densities in the resulting cells [24, 25].

To achieve well-developed ion/electron conduction pathways in the electrodes, it is necessary to inhibit electrode components’ aggregation while enhancing intermolecular interactions between heterogeneous components [26–28]. Previous approaches have primarily centered on the chemical modification of electrode binders [29–32], including the amphiphilic bottlebrush polymers to enhance component dispersion, elastic and composite binders to improve mechanical stability, and polymer-wrapped SWCNTs to establish conductive networks. However, these efforts have often failed to address the complexity that arises from the interactions of multi-components. Furthermore, the intricate multiscaling of binder architecture, coupled with its interactions with carbon conductive additives, has often been overlooked, thereby constraining the dispersion state of the electrode components.

**Fig. 1:**
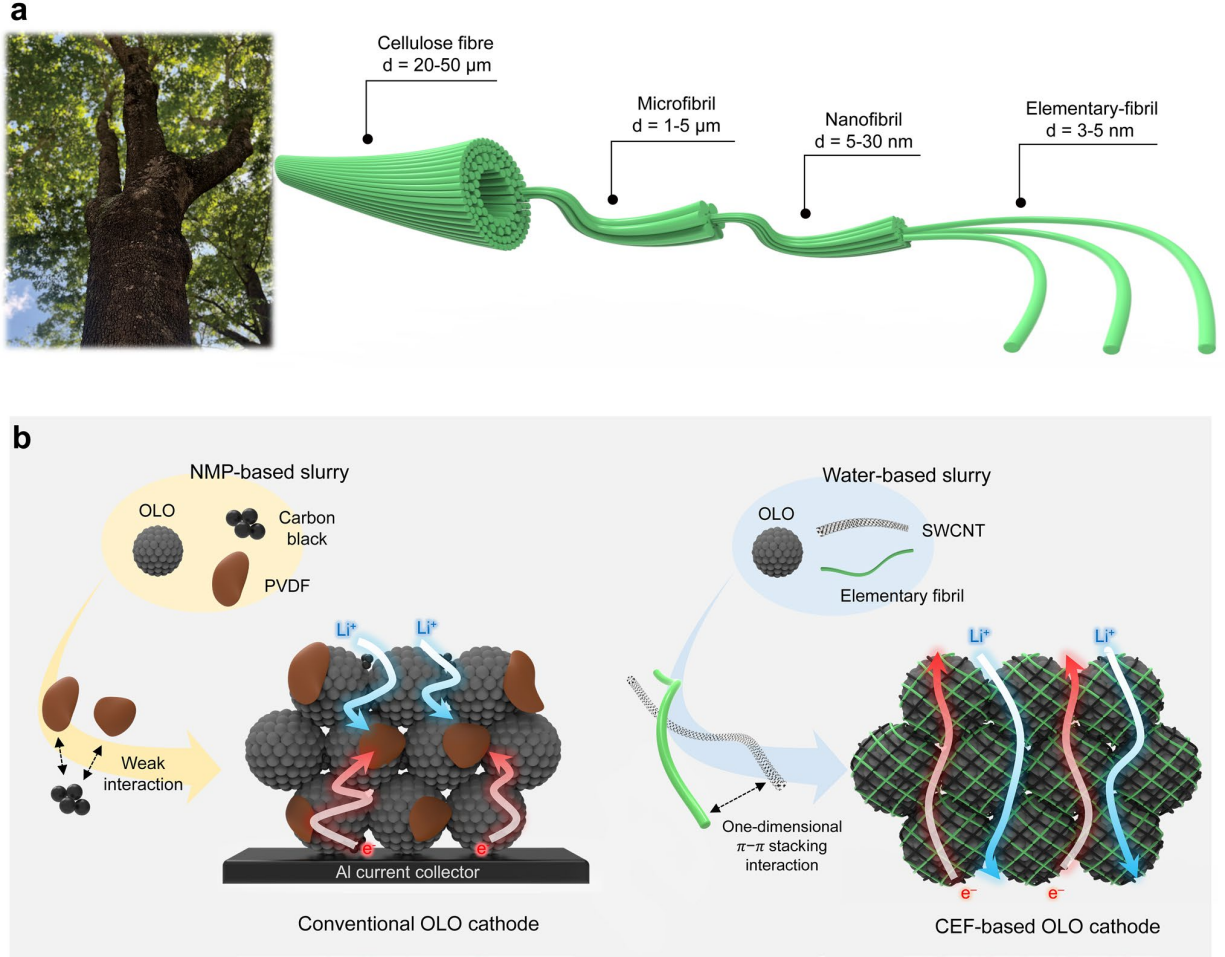
**a** Hierarchical structure of natural wood-derived cellulose. **b** Comparison of dispersion state and ion/electron transport behavior: conventional OLO cathode versus cellulose elementary fibril (CEF)-based OLO cathode

Here, we introduce cellulose elementary fibrils (CEFs) as a new binder to realize high-mass-loading electrodes capable of facilitating the formation of bi-continuous ion/electron conduction networks. CEFs are the finest hierarchical cellulose units with an elementary deagglomerated fibrous configuration. Cellulose nanofibers are irreversibly disintegrated into CEFs by modulating inter- and intramolecular hydrogen bonding between cellulose fibers through the use of a proton-accepting additive and a hydrotropic agent (Fig. 1a) [33–36]. The CEFs, with their increased surface area and anionic surface charge density, enable uniform dispersion with single-walled carbon nanotubes (SWCNTs) via one-dimensional (1D) π-π interaction [37], while retaining their elementary fibrous structure after electrode fabrication. This facilitates the formation of well-connected electron networks throughout the electrode and improves electrolyte accessibility to the electrode active materials. To demonstrate feasibility of the CEF binder, high specific capacity (~ 250 mAh g^–1^) overlithiated layered oxide (OLO) [38, 39] was selected as a model electrode active material. Driven by this structural uniqueness of the electrode enabled by the CEF binder (Fig. 1b), a uniform redox reaction was observed throughout the high-mass-loading OLO cathode. Consequently, the OLO cathode with the CEF binder exhibited the high areal-mass-loading (50 mg cm^–2^, equivalent to 12.5 mAh cm^–2^), allowing the resulting full cell (OLO cathode||Li metal anode) to deliver a high specific energy density (445.4 Wh kg^–1^). These electrochemical metrics far exceeded those achievable with previously reported OLO cathodes based on synthetic polymer binders.

## Experimental Section

### Materials

NaOH (98%), urea (99.5%), ethylene carbonate (EC, 99%), dimethyl carbonate (DMC, 99%), and tris(trimethylsilyl) phosphite (TMSP, 95%) were purchased from Sigma-Aldrich. Li foil (thickness = 100 μm) was purchased from Honjo Chemicals. OLO (0.49Li_2_MnO_3_ᆞ0.51LiNi_0.37_Co_0.24_Mn_0.39_O_2_, average diameter ~ 5 μm), PVDF, and carbon black powders were provided by LG Energy Solution. Pristine CNF and TEMPO-oxidized CNF suspensions were prepared by the National Institute of Forest Science (Korea). SWCNTs (average diameter ~ 1.5 nm) were purchased from Tuball.

### Fabrication of CEFs

The TEMPO-oxidized CNF suspensions (2 wt%) were dispersed into 7 wt% NaOH and 12 wt% urea aqueous solution, precooled to − 10 °C, and stirred for 5 min to obtain a cellulose elementary fibrils (CEFs)-containing aqueous suspension. The obtained suspension was dialyzed with a dialysis tube to remove NaOH and urea.

### Characterization of CEFs and Electrode Slurries

The FT-IR spectra of the electrolytes were recorded with an FT-IR spectrometer (670, Varian). To characterize the deagglomerated state of the CEFs in the aqueous suspension, we conducted transmission electron microscopy (TEM, JEM-2100F, JEOL) analyses. The Raman analysis was conducted with a 514 nm laser (LabRAM HR Evolution Visible_NIR, HORIBA).

### Structural and Physicochemical Characterization of CEF Cathodes

The surface and cross-sectional morphologies of the OLO cathodes were investigated via field emission secondary electron microscopy (FE-SEM, S-4800, Hitachi). The electrical resistivities were measured via a four-point probe technique (CMT-SR1000N, Advanced Instrument Tech). The chemical change of the cathode surface after the cycling test was analyzed by utilizing TOF–SIMS (ION TOF) with a Bi_3_^2+^ gun (25 keV, 1 pA). The ICP-MS (ELAN DRC-2, Perkin Elmer) analysis was conducted to quantitatively estimate the metal (Ni, Co, and Mn) deposited on the Li metal anode after the cycling test. EBAC measurements were performed with an EBIC system (Point Electronic GmbH, Germany) in the FE-SEM (Teneo VS, Thermo Fisher Scientific, USA). A spot size of 14 was utilized at an accelerated voltage of 15 kV.

### Fabrication of Electrodes and Cells and Their Electrochemical Characterizations

For the fabrication of a CEF-based OLO cathode, SWCNTs were mixed with CEF binder at a composition ratio of SWCNT/CEF = 5/5 (w/w) by sonication in water for 10 min, without any dispersion additives. OLO particles were dispersed in a composition ratio of OLO/SWCNT/CEF = 90/5/5 w/w/w. The slurry mixture underwent vacuum-assisted filtration and freeze-drying. A self-standing CEF-based OLO cathode was obtained after being roll-pressed at room temperature and vacuum-dried at 120 °C/12 h. A control OLO cathode was fabricated by casting a slurry mixture of OLO/carbon conductive additives (carbon black or SWCNTs)/PVDF (= 90/5/5, w/w/w) in NMP onto an Al foil, followed by roll-pressing at room temperature and vacuum-drying at 120 °C/12 h. The same dispersion method used for the CEF-based OLO cathode slurry was applied to the control OLO cathode slurry. The ion conductivity of electrodes was estimated by electrochemical impedance spectroscopy (EIS) measurement and analysis of the symmetric cells (electrode|separator|electrode) at a frequency ranging from 10^−2^ to 10^6^ Hz and an applied amplitude of 10 mV utilizing potentiostat/galvanostat (VSP classic, Bio-Logic). The electrochemical performance of OLO cathodes was characterized using a 2032-type coin cell (composed of OLO cathode (areal-mass-loading = 18.4, 22, 30, and 50 mg cm^–2^)||Li metal anode (thickness = 100 μm)). A liquid electrolyte of 1 M LiPF_6_ in EC/DMC = 1/1 (v/v) with 0.5 wt% tris(trimethylsilyl) phosphite (TMSP) was utilized. The galvanostatic intermittent titration technique (GITT) analysis was conducted with an interruption time between each pulse of 1 h. The cell performance was investigated with a cycle tester (PNE Solution) in a chamber set at 25 °C at various charge/discharge conditions.

## Results and Discussion

### Structural Analysis of the CEFs: Focusing on Intermolecular Hydrogen Bonding

**Fig. 2:**
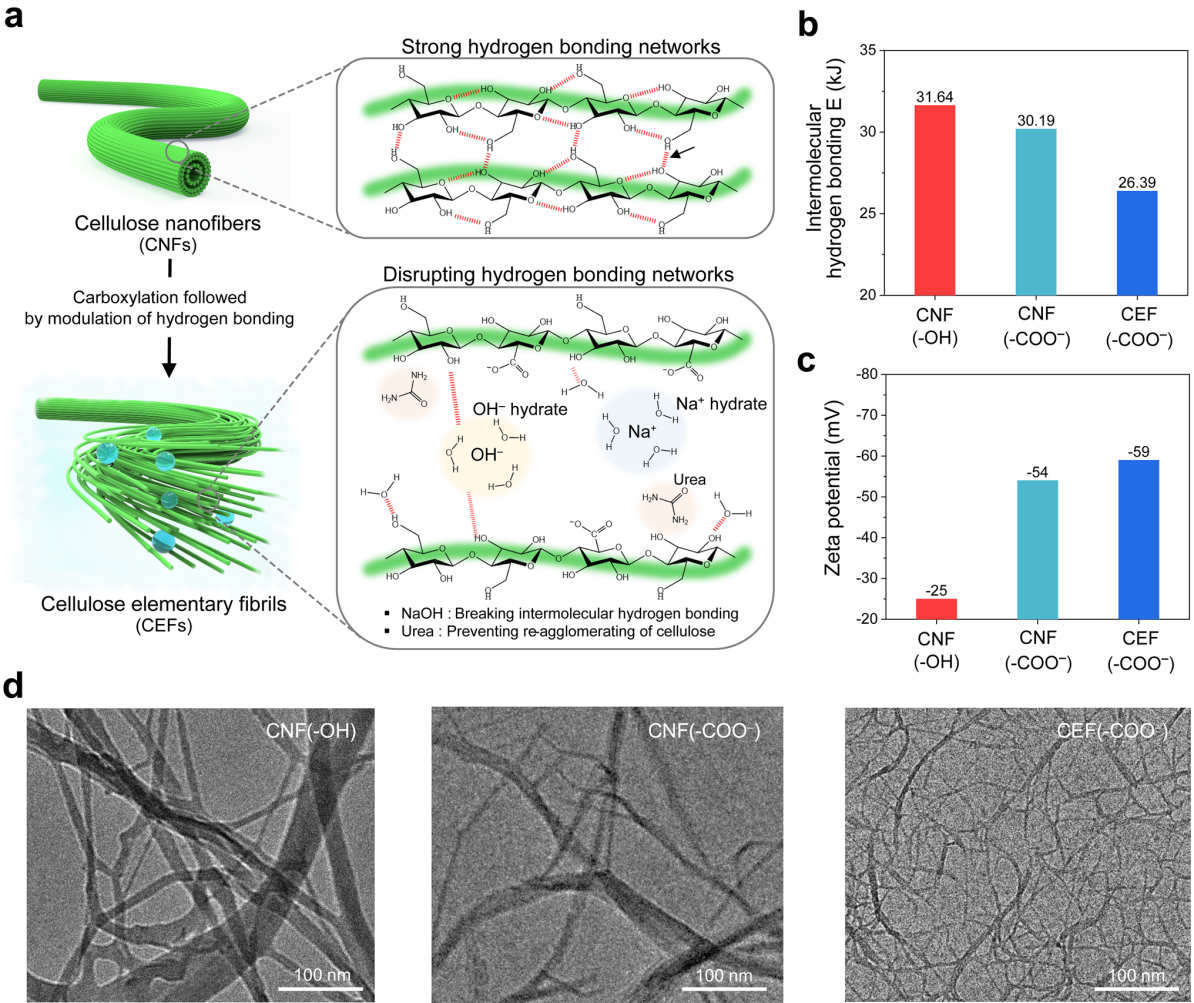
Structural analysis of the CEFs: focusing on intermolecular hydrogen bonding. **a** Schematic of the hierarchical structure and fibrillation mechanism of cellulose. The CEFs were produced from cellulose nanofibers via the carboxylation and the alkali/urea treatment. **b** Intermolecular hydrogen bonding energies calculated from FT-IR spectra of the cellulose aqueous suspensions. **c** Zeta potential of the cellulose aqueous suspensions. **d** TEM images of CNF(–OH), CNF(–COO^–^), and CEF(–COO^–^)

The hierarchical fibrous architecture of cellulose is depicted in Fig. 2a. The cellulose nanofibers (denoted as CNF(–OH)), characterized by diameters ranging from 5 to 30 nm, comprise several elementary fibrils [40]. The strong hydrogen bonding and dense packing between the molecular chains of the CNFs often lead to the formation of larger agglomerates [41]. A common approach implemented to address this issue is the utilization of TEMPO (2,2,6,6-tetramethylpiperidine-1-oxyl) modification to exfoliate the cellulose fibers and increase the concentration of carboxyl groups on their surfaces, thereby generating carboxylated cellulose nanofibers (CNF(–COO^–^)) [42].

To produce CEFs, our strategy involves the modulation of intermolecular hydrogen bonding in cellulose, attempting to maximize cellulose’s surface area while maintaining its inherent fibrous morphology without agglomeration. The hydrogen bonding in cellulose is categorized into two types [43]: intramolecular and intermolecular, with the latter playing a vital role in mitigating the fibril agglomeration. The CNF(–COO^–^) was treated with NaOH (as a proton-accepting additive) and urea (as a hydrotropic agent). This treatment regulated the intermolecular hydrogen bonding, thus enabling the irreversible and deagglomeration of nanofibers into elementary fibrils to form CEF(–COO^–^). Specifically, the NaOH hydrates and free water disrupted the hydrogen bonding networks between the cellulose molecules, facilitating their disintegration. In addition, the urea hydrates formed the hydration layers adjacent to the surface of cellulose molecules [43, 44], which can effectively prevent re-agglomeration of the cellulose molecules.

The intermolecular interactions between different cellulose fibrils (CNFs with hydroxyl groups (CNF(–OH)), CNFs with carboxylate groups (CNF(–COO^–^)), and CEFs with carboxylate groups (CEF(–COO^–^))) were characterized via Fourier transform infrared (FT-IR) spectroscopy, with a focus on hydrogen bonding spectral region (3000–3700 cm^–1^). The spectra of the CEF(–COO^–^) exhibited a downfield shift (~ 3238 cm^–1^) relative to those of the CNF(–OH) and CNF(–COO^–^), indicating a reduction in the intermolecular interactions (Fig. S1) [45].

Employing these FT-IR results, the intermolecular hydrogen bonding energies were estimated quantitatively with Eq. (1) [45, 46]:

1 $$ E_{{\text{H}}} \left( {{\text{kJ}}} \right) = \frac{1}{k} \times \frac{{\upsilon_{0} - \upsilon }}{{\upsilon_{0} }} $$

where $E_{{\text{H}}} { }$ is the hydrogen bonding energy (kJ), $\upsilon_{0}$ is the standard frequency of free OH groups (3600 cm^–1^), $\upsilon$ is the frequency of bound –OH groups (cm^–1^), and $k$ is a constant (*k*^–1^ = 262.5 kJ). The $E_{{\text{H}}} { }$ of CEF(–COO^–^) was calculated to be 26.39 kJ, which is lower than that of CNF(–OH) (31.64 kJ) and CNF(–COO^–^) (30.19 kJ). This result reveals a weakening of the hydrogen bonding networks in the CEFs (Fig. 2b). Despite the insignificant difference in the anionic characteristics of the CNF(–COO^–^) (54 mV) and CEF(–COO^–^) (59 mV) (Fig. 2c), measured by zeta potential analysis, the lower *E*_H_ of the CEF(–COO⁻) demonstrates the critical role of the proton-accepting additive (NaOH) and the hydrotropic agent (urea) in restructuring the intermolecular hydrogen bonding network of cellulose fibers, thereby facilitating the elementary fibrillation.

The high-resolution transmission electron microscopy (HR-TEM) images (Fig. 2d) revealed that the CNF(–OH) has diameters in the hundreds of nanometers with a nonuniform, agglomerated morphology due to uncontrolled intermolecular hydrogen bonding. The CNF(–COO^–^) had smaller diameters because of their surface charges, but some agglomeration between the fibrils remained evident. In contrast, the CEF(–COO^–^) exhibited diameters of only a few nanometers, indicating the weakened intermolecular hydrogen bonding. Consequently, a uniform and deagglomerated fibrous morphology was achieved at the elementary level.

### Effect of the CEF(–COO^–^) Binders on the Dispersion Stability of the Cathode Slurry

**Fig. 3:**
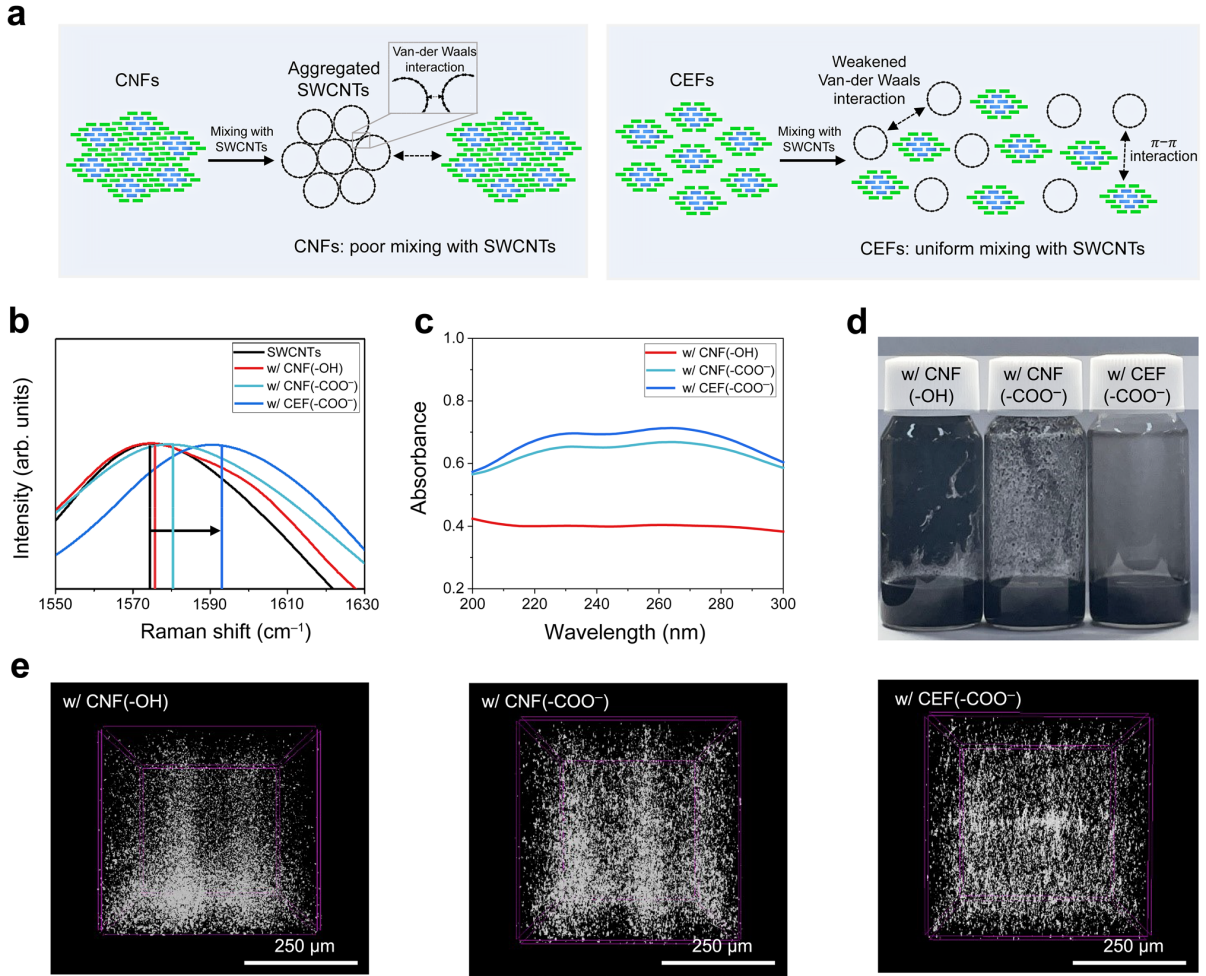
Effect of the CEF(–COO^–^) binders on dispersion stability of the cathode slurry. **a** Schematic of cellulose (CNF vs. CEF) − SWCNT interactions. **b** High-resolution Raman spectra of the cellulose (CNF(–OH) vs. CNF(–COO^–^) vs. CEF(–COO^–^)) aqueous suspensions containing SWCNTs. **c** UV–vis spectra of the cellulose aqueous suspensions containing SWCNTs. **d** Photograph of the cellulose aqueous suspensions containing SWCNTs. **e** Micro-CT images of the aqueous suspensions containing SWCNTs

Cellulose is an amphiphilic polymer with a hydrophobic glucose backbone and hydrophilic hydroxyl side chains [47]. This structural uniqueness may facilitate uniform mixing with carbon nanotubes in aqueous suspensions. However, cellulose fibrils are susceptible to agglomeration if their hydrogen bonding networks are not precisely regulated. This unwanted agglomeration reduces the effective surface area needed for interaction with carbon conductive additives, hindering the formation of a uniform dispersion state (Fig. 3a).

High-resolution Raman spectroscopy was employed to characterize the interactions between cellulose fibrils and SWCNTs in an aqueous suspension (Fig. 3b), in which the same mixing protocol was applied to all suspensions examined herein. The shift in the G-band peak represents van der Waals interactions between the CNTs, indicating the degree of self-aggregation of SWCNTs [48–50]. The Raman spectra of the SWCNTs/CEF(–COO^–^) showed a blue shift from 1573 (pristine SWCNTs) to 1592 cm^−1^, revealing that the effective π-π stacking interactions between the SWCNTs and CEF(-COO^–^) led to the de-aggregation of the SWCNTs. In contrast, relatively insignificant shifts were detected with the CNF(–OH) (1574 cm^–1^) and CNF(–COO^–^) (1579 cm^–1^), indicating their limited interactions with the SWCNTs. This is due to the uncontrolled agglomeration of the CNFs, which is insufficient to provide the effective surface area available for dispersion of the SWCNTs.

The dispersion stability of the SWCNT suspensions incorporating different cellulose fibrils was analyzed via ultraviolet–visible (UV–vis) spectroscopy after centrifugation (Fig. 3c). The absorbance intensity of the spectra increased in the order of CNF(–OH), CNF(–COO^–^), and CEF(-COO^–^). This result indicates that the SWCNT suspensions with CNF(–OH) and CNF(-COO^–^) binders showed faster sedimentation kinetics, revealing the formation of relatively larger SWCNT aggregates [21]. Consequently, these poorly dispersed suspensions yielded the clear upper layers after centrifugation. The upper layers of the SWCNT suspensions with CNF(–OH) and CNF(–COO^–^) binder showed lower absorbance intensities due to a reduced fraction of individually dispersed SWCNTs. In contrast, the higher absorbance intensity in the upper layer of the SWCNT suspension with CEF(–COO^–^) binder reflected a well-dispersed state of the SWCNTs. These results were confirmed by visual comparison of the aqueous suspensions stored for 12 h after mechanical mixing for 10 min (Fig. 3d). In this static sedimentation test, the suspension with the CEF(–COO^–^) demonstrated a superior dispersion state, devoid of agglomeration, whereas the suspensions with the CNF(–COO^–^) and CNF(-OH) showed severe aggregation of SWCNTs adhering to the bottle walls. The microstructure of these suspensions was further elucidated via micro-computed tomography (Micro-CT) analysis (Fig. 3e). The uniform dispersion of the SWCNTs was observed in the suspension with the CEF(–COO^–^), whereas the aggregates of the particle mixtures were noticeable in the suspensions with the CNF(–OH) and CNF(–COO^–^). This result underscores the viable role of the CEF binder in achieving a well-dispersed cathode slurry.

### Electrochemical Performance of the OLO Cathodes

**Fig. 4:**
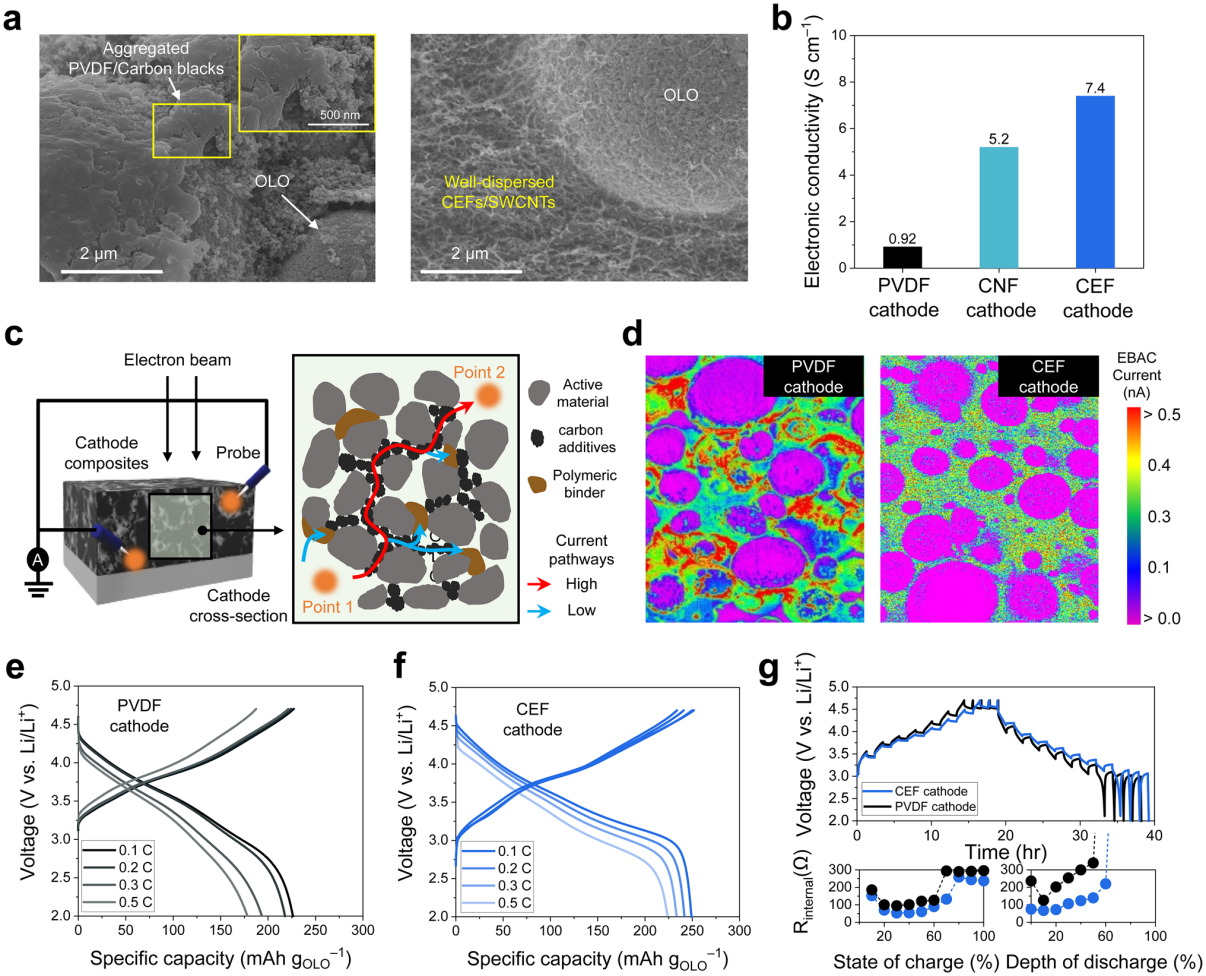
Electrochemical performance of the OLO cathodes. **a** SEM images of the (left) PVDF and (right) CEF cathodes. **b** Electronic conductivities of the PVDF, CNF, and CEF cathodes. **c** Schematic depicting EBAC analysis of the OLO cathodes. **d** Spatial distribution of electronic resistances of the (left) PVDF and (right) CEF cathodes utilizing EBAC analysis. **e**–**f** Charge/discharge voltage profiles of the PVDF and CEF cathodes at varied discharge current rates (0.1C (= 0.55 mA cm^–2^)–0.5C (= 2.75 mA cm^–2^)) at a fixed charge current rate of 0.1C. **g** (top) GITT profiles upon repeated current stimuli at charge/discharge current rate of 0.1C/0.1C (= 0.55 mA cm^–2^) and (bottom) internal cell resistance (*R*_internal_) as a function of SOC and DOD

To investigate the effect of the dispersion state of the carbon conductive additives and binders on the structural integrity of OLO cathodes, three different cathodes with conventional polyvinylidene fluoride (PVDF), CNF(–COO^–^), and CEF(–COO^–^) binders were fabricated at a composition ratio of OLO/binder/carbon conductive additives (carbon black powders for the PVDF binder, and SWCNTs for the CNF(–COO^–^) and CEF(–COO^–^) binders) = 90/5/5 (w/w/w). The OLO cathodes with CNF(–COO^–^) and CEF(–COO^–^) binders were fabricated using a vacuum-assisted filtration process, yielding a self-standing and flexible electrode (Fig. S2). The control PVDF cathode with similar areal-mass-loading was fabricated via a typical slurry-casting method. The surface scanning electron microscopy (SEM) images of the obtained electrodes (Figs. 4a and S3) showed the aggregation of the binder/carbon conductive additives mixtures in the OLO cathodes with the PVDF and CNF (–COO^–^) binders. In contrast, the OLO cathode with the CEF(–COO^–^) exhibited the uniformly dispersed CEF/SWCNTs mixtures owing to the effective intermolecular π-π interaction, facilitating the formation of continuous electronic conduction networks. This result was verified by measuring the electronic conductivities of the OLO cathodes using the four-point probe analysis (Fig. 4b). The OLO cathode with the CEF (–COO^–^) (referred to as CEF cathode) exhibited a higher electronic conductivity (7.4 S cm^–1^), whereas the lower electronic conductivities were observed at the OLO cathode with the CNF(–COO^–^) (referred to as CNF cathode) (5.2 S cm^–1^) and the OLO cathode with the PVDF (0.92 S cm^–1^).

The spatial distribution of electron conduction channels in the OLO electrodes was investigated using electron beam-absorbed current (EBAC) analysis (Fig. 4c). The probed current signal enables the mapping of current pathways (electron conduction channels), identifying the regions of high electrical resistance [51]. The EBAC images of the electrode cross-sections (Fig. 4d) exhibited that the current signal is influenced by the electrical properties of the electrode components such as OLO, carbon conductive additives, and polymer binders. The EBAC image of the PVDF cathode displayed the pronounced localization of electron conduction channels, with severe heterogeneity attributed to electrically isolated regions resulting from the random aggregation of the binder/carbon black powders mixtures. In contrast, the CEF cathode exhibited uniformly distributed electron conduction channels owing to the homogeneous dispersion of SWCNTs, which was enhanced by the optimized intermolecular interactions between the CEF binders and SWCNTs. Meanwhile, the EBAC analysis of the CNF cathode revealed the formation of relatively inhomogeneous electron conduction pathways (Fig. S4a), although its overall electronic conductivity appeared similar to that of the CEF cathode. Consequently, the CNF cathode showed the unsatisfactory electrochemical performance compared to the CEF cathode (Fig. S4b–d), further underscoring the importance of elementary fibrillation in achieving uniform dispersion of SWCNTs.

In addition, the homogeneous distribution of electrode components can facilitate ion transport in the electrodes. To investigate the ion transport phenomena in the CEF cathode, electrochemical impedance spectroscopy (EIS) measurement was conducted with a blocking symmetric cell (electrode||electrode) at 0% state of charge (SOC) (Fig. S5). The projection of a slope (observed in the low-frequency region of the complex impedance plot) to a real axis, defined as the ionic resistance (*R*_ion_)/3, reflects the ionic resistance inside the cathodes [52]. The CEF cathode exhibited a lower *R*_ion_/3 (~ 1.22 Ω cm^2^) than the PVDF cathode (~ 5.84 Ω cm^2^). This lower ionic resistance of the CEF cathode was attributed to the formation of well-connected ion conduction channels resulting from the homogeneous dispersion of SWCNTs and CEF binder within the cathode. The structural difference between the CEF and PVDF cathodes is previously investigated in Fig. 4a, b. Compared to the PVDF cathode, which showed the nonuniform distribution of the electrode components (resulting in a poorly developed porous structure that will be filled with electrolytes) along the through-thickness direction, the CEF cathode exhibited the homogeneous distribution of electrode components, beneficially contributing to the formation of highly interconnected ion conduction channels in the through-thickness direction.

The charge/discharge rate capabilities of the CEF and PVDF cathodes, both with the same areal-mass-loading of 22 mg cm^−2^, were evaluated by varying the charge/discharge current rates from 0.1 C (= 0.55 mA cm^−2^) to 0.5 C (= 2.75 mA cm^−2^). OLO active materials have a high theoretical capacity of 250 mAh g_OLO_^–1^, but their redox reaction kinetics is hindered by low electronic conductivity [38]. The PVDF cathode failed to realize the theoretical capacity of OLO, mainly because of the poorly developed electron conduction channels (shown in Fig. 4d), resulting in a low discharge capacity of 228 mAh g_OLO_^−1^ at 0.1C (Fig. 4e). With increasing current rates, the discharge capacity of the PVDF cathode decreased significantly to 178 mAh g_OLO_^–1^ because of the increased ohmic polarization. To ensure a fair comparison, a PVDF cathode containing the same SWCNTs as the CEF-based OLO cathode was prepared as another control sample. The resulting PVDF cathode exhibited severe cracking after solvent drying, indicating unwanted SWCNT aggregation. Further work is required to address the dispersion issues associated with SWCNTs in the preparation of PVDF-based OLO cathodes. Despite this structural instability (Fig. S6), we carefully selected cathode samples with minimal cracking and then evaluated their electrochemical performance. The PVDF cathode with SWCNTs demonstrated very low discharge capacity and poor cycle life, which was further corroborated by significant aggregation of electrode components due to their poor dispersibility (Fig. S7). This finding highlights the importance of achieving a uniform dispersion state for electrochemically reliable electrodes, particularly when incorporating high-aspect-ratio conductive additives such as SWCNTs. In contrast, the CEF cathode provided a high specific discharge capacity of 250 mAh g_OLO_^−1^ at 0.1C under this high areal-mass-loading of 22 mg cm^−2^, which was close to the theoretical capacity (~ 250 mAh g_OLO_^−1^) of OLO (Fig. 4f). The galvanostatic intermittent titration technique (GITT) analysis performed during charging/discharging of the cells revealed that the CEF cathode effectively alleviated the rise in cell polarization upon repeated current stimuli (applied at 0.1 C (= 0.55 mA cm^−2^) and interruption time between the pulses of 1 h), wherein the obtained internal cell resistances (*R*_internal_) were presented as a function of SOC and depth of discharge (DOD) (Fig. 4g). Additionally, the CEF cathode exhibited higher Li^+^ diffusion coefficient (4.23 × 10^–9^ cm^2^ s^–1^) than the PVDF cathode (3.29 × 10^–9^ cm^2^ s^–1^), indicating the facile Li^+^ transport in the CEF cathode (Fig. S8 and Table S1).

### Superior Cycle Life of the CEF Cathode over the PVDF Cathode

**Fig. 5:**
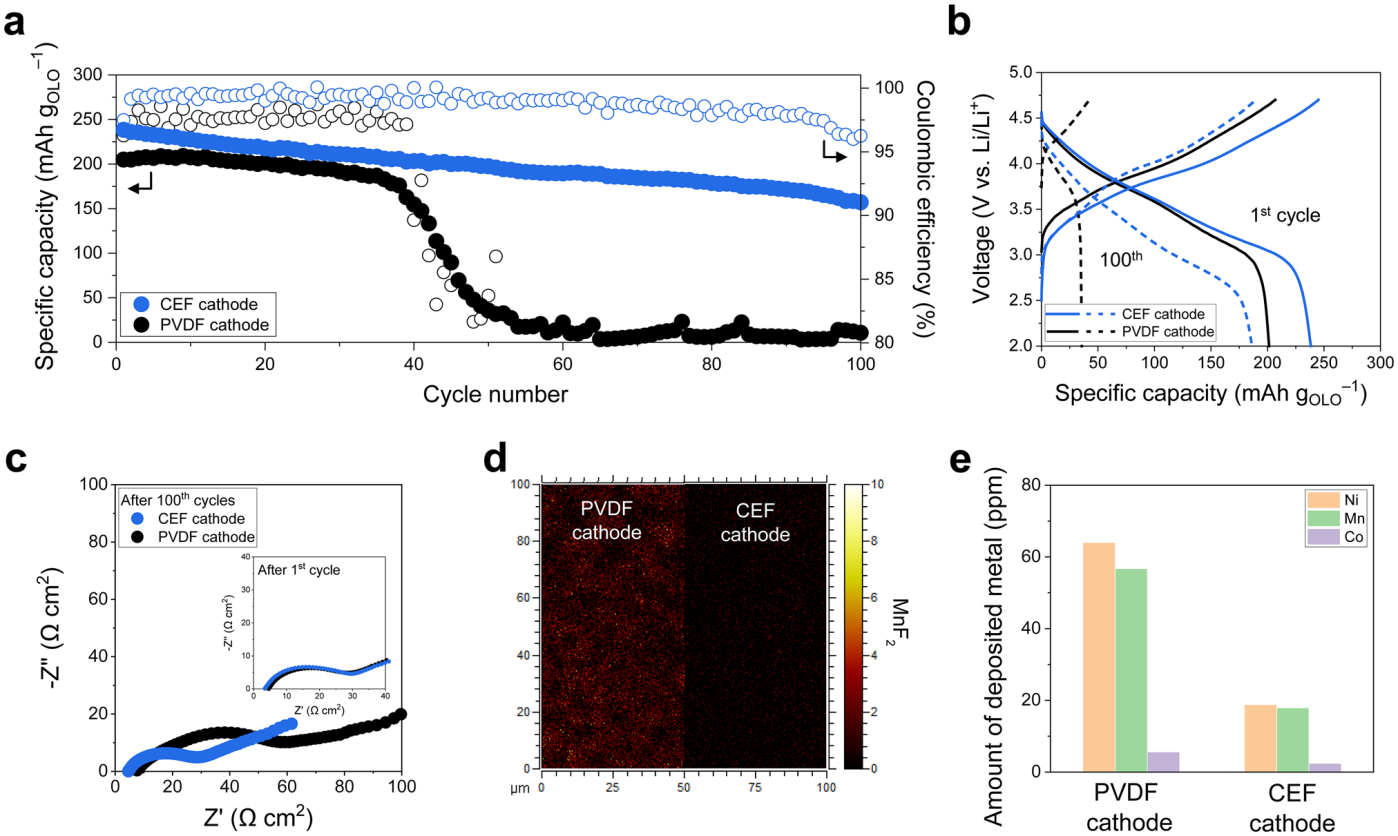
Cycling performance of the cells (OLO cathode (areal-mass-loading = 18.4 mg cm^–2^)||Li metal anode (100 μm)). **a** Cycling retention of the cells at charge/discharge current rates of 0.2C/0.2C (= 0.92 mA cm^–2^/0.92 mA cm^–2^) under a voltage range of 2.0–4.7 V. **b** Charge/discharge voltage profiles of the cells at 1st and 100th cycle. **c** EIS profiles of the cells after 1st cycle (inset) and 100 cycles. **d** TOF–SIMS mapping images of the MnF_2_ byproducts formed on the surface of cycled OLO cathodes. **e** Amount of metallic Ni, Mn and Co deposited on cycled Li metal anodes via ICP-MS analysis

A Li metal full cell (OLO cathode (areal-mass-loading = 18.4 mg cm^–2^)||Li metal anode (thickness = 100 μm), a liquid electrolyte of 1 M LiPF_6_ in EC/DMC = 1/1 (v/v) with a 0.5 wt% tris(trimethylsilyl) phosphite (TMSP) additive) with the PVDF cathode showed only 15% capacity retention after 60 cycles at a charge/discharge current rate of 0.2C/0.2C (= 0.92 mA cm^−2^/0.92 mA cm^−2^) (Fig. 5a, b). Meanwhile, the fluctuation of the coulombic efficiency was observed, which could stem from the use of OLO active materials. It is known that OLO active materials are promising due to their high theoretical capacity and operating voltage. However, they often show unstable and low coulombic efficiency, mainly due to the severe transition metal dissolution and structural degradation, even at low areal-mass-loadings [53, 54]. Dissolved transition metal ions (including Mn^2^⁺, Co^2^⁺, and Ni^2^⁺) cause unwanted passivation of Li metal anodes, thereby accelerating cell degradation [55–57]. In contrast, the CEF cathode exhibited higher capacity retention during cycling (80% after 100 cycles). The improved cycling performance of the CEF cathode was verified by examining the change in the cell resistance after the cycling test. The CEF cathode significantly suppressed the cell resistance growth compared to the PVDF cathode (Fig. 5c).

During the cycling test, transition metal ions such as Ni^2+^, Co^2+^, and Mn^2+^ tend to leach from the OLO cathode materials into electrolytes [58]. These dissolved transition metal ions subsequently react with hydrofluoric acid (HF, typically generated in the LiPF_6_-based liquid electrolyte), forming unwanted byproducts on the cathode surface. They also migrate through separator membranes and deposit on Li metal anodes as passivation layers [59, 60]. The time-of-flight secondary ion mass spectrometry (TOF–SIMS) mapping images of the cycled cathodes indicated that the formation of MnF_2_ byproducts was mitigated on the CEF cathode compared to the PVDF cathode (Fig. 5d). To better understand this advantageous effect of the CEF cathode, a model study was conducted, in which the CEF binder was immersed in carbonate (EC/DMC = 1/1 (v/v))-based electrolytes containing Mn^2+^ as well as Li^+^ and PF_6_^−^ (Fig. S9). These solutions were stored at 45 °C for 5 days to expedite possible side reactions. The simultaneous presence of Mn^2+^ and PF_6_^–^ in the electrolytes accelerated the parasitic reactions, as evidenced by a visible color change (Fig. S9b). In contrast, color change was not observed in the electrolyte containing the CEF, demonstrating that the CEF was effective in suppressing Mn^2+^-induced side reactions. In addition, the inductively coupled plasma mass spectroscopy (ICP-MS) analysis confirmed that the contamination of Li metal anodes by the deposition of metallic Ni, Co, and Mn was significantly reduced by the CEF cathode more than the PVDF cathode (Fig. 5e). Moreover, the Li metal anode paired with the CNF cathode was more contaminated than the Li metal anode coupled with the CEF cathode, underscoring the importance of reducing cellulose fibril agglomeration (Fig. S10). These results indicate that the CEF binder, driven by its anionic (COO^–^) feature that enables intermolecular electrostatic attraction, played a viable role in chelating the transition metal ions dissolved from the OLO, thereby potentially suppressing further Mn^2^⁺ dissolution according to the Le Chatelier’s principle [61]. Consequently, the cycle life of the resulting cell can be extended.

### Contribution of the CEF Binder to High-Mass-Loading OLO Cathodes

**Fig. 6:**
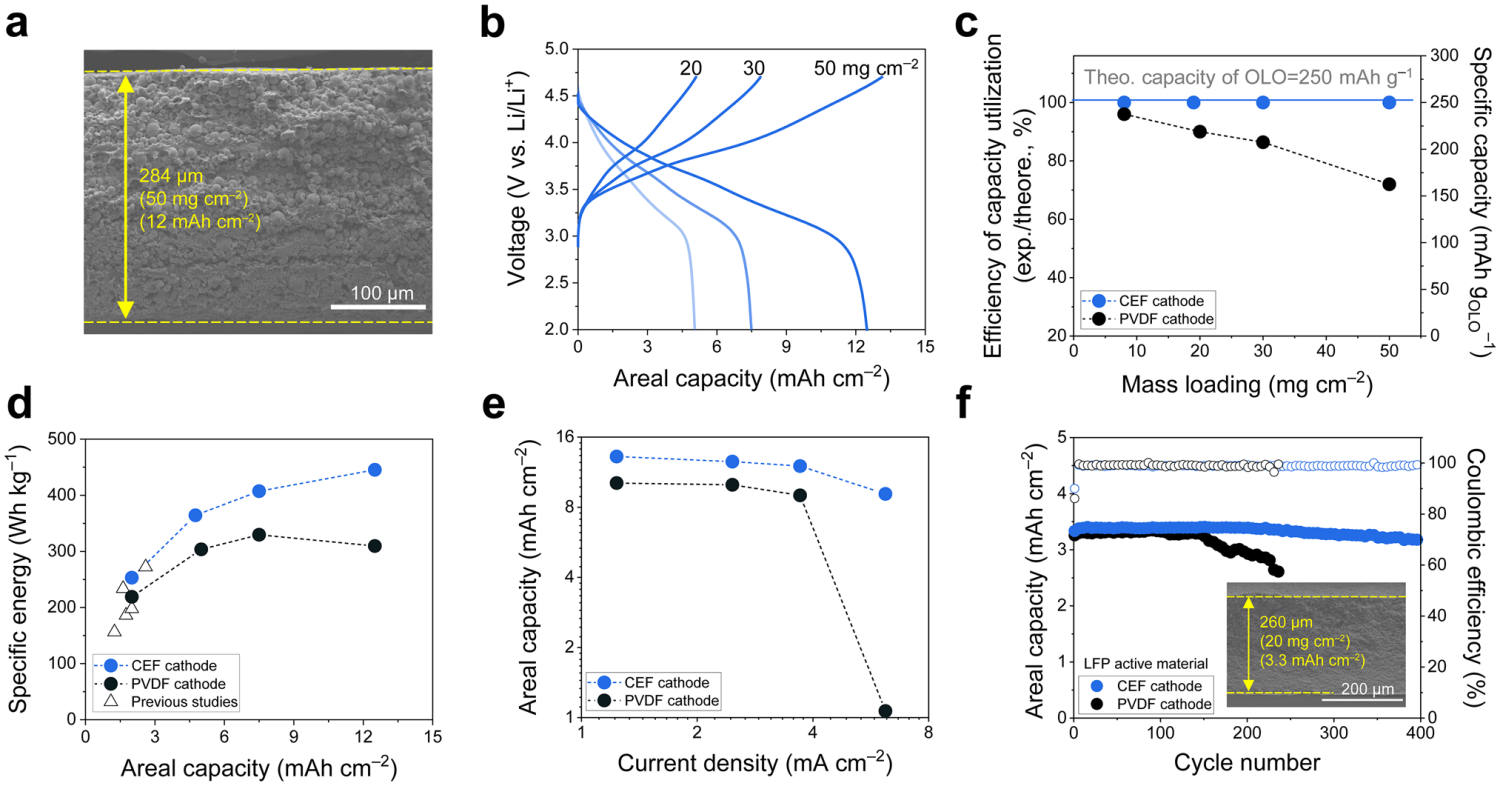
Contribution of the CEF binder to high-mass-loading OLO cathodes. **a** Cross-sectional SEM image of the CEF cathode (areal-mass-loading = 50 mg cm^–2^). **b** Charge/discharge voltage profiles of the cells as a function of areal-mass-loading of the CEF cathodes at charge/discharge current rates of 0.05C/0.1C and voltage range of 2.0–4.7 V. **c** Specific capacity retention of the PVDF and CEF cathodes as a function of areal-mass-loading. **d** Specific energies (calculated based on the entire weight of the cells) as a function of areal capacity (PVDF cathode vs. CEF cathode vs. previously reported OLO cathodes). **e** Discharge rate performance of the PVDF and CEF cathodes (areal-mass-loading = 50 mg cm^–2^) at a fixed charge current rate of 0.05C. **f** Cycling retention of the LFP cells with an areal-mass-loading of 20 mg cm^–2^ at charge/discharge current densities of 0.5C/1.0C (= 1.65 mA cm^–2^/3.3 mA cm_–2_) and voltage range of 2.5–3.7 V. Cross-sectional SEM image of the CEF LFP cathode (inset)

Realizing the theoretical capacities of electrode active materials is critical to developing high-energy–density cells and is particularly challenging for high-mass-loading electrodes. Despite its high theoretical capacity, OLO has encountered limitations in high-mass-loading applications due to structural instability and low electronic conductivity (~ 10^–9^ S cm^–1^) [62]. CEF cathodes were fabricated with varying mass loadings of 20, 30, and 50 mg cm^−2^. The cross-sectional SEM image of the CEF cathode (284 μm, corresponding to 50 mg cm^−2^) depicted a uniform distribution of the cathode components in the through-thickness direction (Fig. 6a). The effect of the CEF cathodes on the electrochemical performance of the full cells was investigated at a charge/discharge current rate of 0.05C/0.1C.

The CEF cathodes exhibited higher areal discharge capacities than the PVDF cathodes over the entire range of areal-mass-loadings examined herein (Figs. 6b andS11). Moreover, the specific capacity of OLO in the CEF cathodes remained almost constant at 250 mAh g_OLO_^−1^ together with stable cycling performance even at the high areal-mass-loadings of 30 and 50 mg cm^−2^ (Fig. S12). In contrast, the PVDF cathodes exhibited a considerable decrease in the specific capacity with increasing areal-mass-loadings (Fig. 6c). This result indicates that the CEF cathodes, driven by their well-established ion/electron conduction channels, enabled the full realization of the theoretical specific capacity of OLO, which can eventually contribute to the achievement of high cell energy densities [63]. Furthermore, the CEF cathode-containing cells allowed a continuous increase in the specific energy, increasing the areal capacity. In comparison, the PVDF cathode-containing cells reached a peak specific energy at an areal capacity of 7.5 mAh cm^–2^, and there was a decline in specific energy as the areal capacity was further increased to 12.5 mAh cm^–2^ owing to the insufficient utilization of the specific capacity of OLO (Fig. 6d). Here, the specific energies of the cells were estimated without including packaging substances (see Table S2 for calculation details), in which the absence of heavy metallic foil current collectors is an additional contribution to the higher specific energies of the cells [64–67]. In particular, the CEF cathode-containing cell achieved a specific energy of 445.4 Wh kg^−1^ with an areal capacity of 12.5 mAh cm^−2^, far exceeding those of previously reported high-mass-loading OLO cells (Fig. 6d and Table S3). The CEF cathode with an areal-mass-loading of 50 mg cm⁻^2^ demonstrated stable charge/discharge behavior while delivering the theoretical capacity of OLO (~ 250 mAh g^−1^) in pouch-type configuration, highlighting its potential for large-scale applications (Fig. S13) [68]. In addition, the CEF cathodes exhibited a higher discharge rate performance than the PVDF cathodes (Fig. 6e), verifying the formation of well-interconnected ion/electron conduction networks inside the high-mass-loading (50 mg cm^−2^) OLO cathodes.

The CEF binder was combined with LiFePO_4_ (LFP) active materials to explore its potential application to other electrode materials. A high areal-mass-loading (~ 20 mg cm^–2^) LFP cathode with the CEF binder exhibited a stable cycle life and superior capacity retention compared to the PVDF cathode (Fig. 6f), demonstrating the viable role and versatility of the CEF binder.

## Conclusions

In summary, CEFs were presented as an elementary deagglomerated binder for high-mass-loading electrodes. The CEFs were prepared by modulating the intermolecular hydrogen bonding of cellulose via treatment with NaOH (proton acceptor) and urea (hydrotropic agent). Owing to the finest cellulose unit, the CEFs allowed an increase in the surface area and charge density, facilitating uniform mixing with the SWCNTs. Furthermore, the elemental fibril structure of the CEFs enhanced the electrolyte access to the OLO in the cathode. This architectural uniqueness promoted the formation of well-developed ion/electron conduction networks, ensuring the homogeneous redox reaction throughout the cathode. The anionic surface charge of the CEF binder effectively chelated the transition metal ions dissolved from the OLO via the intermolecular electrostatic attraction, thus stabilizing both the OLO cathode and Li metal anode. Consequently, the CEF cathode achieved a high areal-mass-loading level (50 mg cm^−2^) while maintaining the theoretical specific capacity of OLO over a wide range of mass loadings. Moreover, the CEF binder enabled the OLO cathode-containing Li metal full cell to achieve a specific energy density of 445.4 Wh kg^−1^, outperforming those of previously reported OLO cathodes based on synthetic polymer binders. This CEF strategy provides a new insight into binder design for high-mass-loading electrodes and holds promise as a versatile platform technology applicable to various cathode active materials.

## Supplementary Information

Below is the link to the electronic supplementary material.

Supplementary file1 (DOCX 2896 KB)

## References

[1] R. Schmuch, R. Wagner, G. Hörpel, T. Placke, M. Winter, Performance and cost of materials for lithium-based rechargeable automotive batteries. Nat. Energy **3**, 267–278 (2018). 10.1038/s41560-018-0107-2

[2] J. Lu, Z. Chen, Z. Ma, F. Pan, L.A. Curtiss et al., The role of nanotechnology in the development of battery materials for electric vehicles. Nat. Nanotechnol. **11**, 1031–1038 (2016). 10.1038/nnano.2016.207 27920438 10.1038/nnano.2016.207

[3] J.W. Choi, D. Aurbach, Promise and reality of post-lithium-ion batteries with high energy densities. Nat. Rev. Mater. **1**, 16013 (2016). 10.1038/natrevmats.2016.13

[4] F. Wu, J. Maier, Y. Yu, Guidelines and trends for next-generation rechargeable lithium and lithium-ion batteries. Chem. Soc. Rev. **49**, 1569–1614 (2020). 10.1039/c7cs00863e 32055806 10.1039/c7cs00863e

[5] M. Li, J. Lu, Z. Chen, K. Amine, 30 years of lithium-ion batteries. Adv. Mater. **30**, 1800561 (2018). 10.1002/adma.201800561 10.1002/adma.201800561 29904941

[6] J. Wu, X. Zhang, Z. Ju, L. Wang, Z. Hui et al., From fundamental understanding to engineering design of high-performance thick electrodes for scalable energy-storage systems. Adv. Mater. **33**, e2101275 (2021). 10.1002/adma.202101275 34028911 10.1002/adma.202101275

[7] Y. Kuang, C. Chen, D. Kirsch, L. Hu, Thick electrode batteries: principles, opportunities, and challenges. Adv. Energy Mater. **9**, 1901457 (2019). 10.1002/aenm.201901457

[8] J. Kong, H. Yang, X. Guo, S. Yang, Z. Huang et al., High-mass-loading porous Ti_3_C_2_T_*x*_ films for ultrahigh-rate pseudocapacitors. ACS Energy Lett. **5**, 2266–2274 (2020). 10.1021/acsenergylett.0c00704

[9] M. Singh, J. Kaiser, H. Hahn, A systematic study of thick electrodes for high energy lithiumionbatteries. J. Electroanal. Chem. **782**, 245–249 (2016). 10.1016/j.jelechem.2016.10.040

[10] J. Li, N. Sharma, Z. Jiang, Y. Yang, F. Monaco et al., Dynamics of particle network in composite battery cathodes. Science **376**, 517–521 (2022). 10.1126/science.abm8962 35482882 10.1126/science.abm8962

[11] Z. Jiang, J. Li, Y. Yang, L. Mu, C. Wei et al., Machine-learning-revealed statistics of the particle-carbon/binder detachment in lithium-ion battery cathodes. Nat. Commun. **11**, 2310 (2020). 10.1038/s41467-020-16233-5 32385347 10.1038/s41467-020-16233-5 PMC7210251

[12] X. Zhang, Z. Ju, Y. Zhu, K.J. Takeuchi, E.S. Takeuchi et al., Multiscale understanding and architecture design of high energy/power lithium-ion battery electrodes. Adv. Energy Mater. **11**, 2000808 (2021). 10.1002/aenm.202000808

[13] M. Zhu, J. Park, A.M. Sastry, Particle interaction and aggregation in cathode material of Li-ion batteries: a numerical study. J. Electrochem. Soc. **158**, A1155 (2011). 10.1149/1.3625286

[14] Y. Shi, J. Zhang, A.M. Bruck, Y. Zhang, J. Li et al., A tunable 3D nanostructured conductive gel framework electrode for high-performance lithium ion batteries. Adv. Mater. **29**, 1603922 (2017). 10.1002/adma.201603922 10.1002/adma.201603922 28328016

[15] A. Kraytsberg, Y. Ein-Eli, Conveying advanced Li-ion battery materials into practice the impact of electrode slurry preparation skills. Adv. Energy Mater. **6**, 1600655 (2016). 10.1002/aenm.201600655

[16] K. Ngamchuea, K. Tschulik, S. Eloul, R.G. Compton, *In situ* detection of particle aggregation on electrode surfaces. ChemPhysChem **16**, 2338–2347 (2015). 10.1002/cphc.201500168 26036818 10.1002/cphc.201500168

[17] Z. Fan, J. Yan, L. Zhi, Q. Zhang, T. Wei et al., A three-dimensional carbon nanotube/graphene sandwich and its application as electrode in supercapacitors. Adv. Mater. **22**, 3723–3728 (2010). 10.1002/adma.201001029 20652901 10.1002/adma.201001029

[18] O.V. Kharissova, B.I. Kharisov, E.G. de Casas Ortiz, Dispersion of carbon nanotubes in water and non-aqueous solvents. RSC Adv. **3**, 24812–24852 (2013). 10.1039/C3RA43852J

[19] Y.Y. Huang, E.M. Terentjev, Dispersion of carbon nanotubes: mixing, sonication, stabilization, and composite properties. Polymers **4**, 275–295 (2012). 10.3390/polym4010275

[20] J.-H. Ha, S.-E. Lee, S.-H. Park, Effect of dispersion by three-roll milling on electrical properties and filler length of carbon nanotube composites. Materials (Basel) **12**, 3823 (2019). 10.3390/ma12233823 31766359 10.3390/ma12233823 PMC6926710

[21] L. Jiang, L. Gao, J. Sun, Production of aqueous colloidal dispersions of carbon nanotubes. J. Colloid Interface Sci. **260**, 89–94 (2003). 10.1016/S0021-9797(02)00176-5 12742038 10.1016/s0021-9797(02)00176-5

[22] M. Ganß, B.K. Satapathy, M. Thunga, R. Weidisch, P. Pötschke et al., Structural interpretations of deformation and fracture behavior of polypropylene/multi-walled carbon nanotube composites. Acta Mater. **56**, 2247–2261 (2008). 10.1016/j.actamat.2008.01.010

[23] R. Rastogi, R. Kaushal, S.K. Tripathi, A.L. Sharma, I. Kaur et al., Comparative study of carbon nanotube dispersion using surfactants. J. Colloid Interface Sci. **328**, 421–428 (2008). 10.1016/j.jcis.2008.09.015 18848704 10.1016/j.jcis.2008.09.015

[24] J.-H. Kim, J.-M. Kim, S.-K. Cho, N.-Y. Kim, S.-Y. Lee, Redox-homogeneous, gel electrolyte-embedded high-mass-loading cathodes for high-energy lithium metal batteries. Nat. Commun. **13**, 2541 (2022). 10.1038/s41467-022-30112-1 35534482 10.1038/s41467-022-30112-1 PMC9085813

[25] J.H. Kim, K.M. Lee, J.W. Kim, S.H. Kweon, H.S. Moon et al., Regulating electrostatic phenomena by cationic polymer binder for scalable high-areal-capacity Li battery electrodes. Nat. Commun. **14**, 5721 (2023). 10.1038/s41467-023-41513-1 37714895 10.1038/s41467-023-41513-1 PMC10504278

[26] J.-H. Lee, S.-B. Wee, M.-S. Kwon, H.-H. Kim, J.-M. Choi et al., Strategic dispersion of carbon black and its application to ink-jet-printed lithium cobalt oxide electrodes for lithium ion batteries. J. Power. Sour. **196**, 6449–6455 (2011). 10.1016/j.jpowsour.2011.03.041

[27] G. Liu, H. Zheng, X. Song, V.S. Battaglia, Particles and polymer binder interaction: a controlling factor in lithium-ion electrode performance. J. Electrochem. Soc. **159**, A214–A221 (2012). 10.1149/2.024203jes

[28] H. Qi, J. Liu, S. Gao, E. Mäder, Multifunctional films composed of carbon nanotubes and cellulose regenerated from alkaline–urea solution. J. Mater. Chem. A **1**, 2161–2168 (2013). 10.1039/C2TA00882C

[29] N.-Y. Kim, J. Moon, M.-H. Ryou, S.-H. Kim, J.-H. Kim et al., Amphiphilic bottlebrush polymeric binders for high-mass-loading cathodes in lithium-ion batteries. Adv. Energy Mater. **12**, 2102109 (2022). 10.1002/aenm.202102109

[30] B. Chang, J. Kim, Y. Cho, I. Hwang, M.S. Jung et al., Highly elastic binder for improved cyclability of nickel-rich layered cathode materials in lithium-ion batteries. Adv. Energy Mater. **10**, 2001069 (2020). 10.1002/aenm.202001069

[31] L. Rao, X. Jiao, C.-Y. Yu, A. Schmidt, C. O’Meara et al., Multifunctional composite binder for thick high-voltage cathodes in lithium-ion batteries. ACS Appl. Mater. Interfaces **14**, 861–872 (2022). 10.1021/acsami.1c19554 34964595 10.1021/acsami.1c19554

[32] J.M. Kim, S.H. Kim, N.Y. Kim, M.H. Ryou, H. Bae et al., Nanofibrous conductive binders based on DNA-wrapped carbon nanotubes for lithium battery electrodes. iScience **23**, 101739 (2020). 10.1016/j.isci.2020.101739 33235982 10.1016/j.isci.2020.101739 PMC7670196

[33] T. Li, C. Chen, A.H. Brozena, J.Y. Zhu, L. Xu et al., Developing fibrillated cellulose as a sustainable technological material. Nature **590**, 47–56 (2021). 10.1038/s41586-020-03167-7 33536649 10.1038/s41586-020-03167-7

[34] D. Sawada, Y. Nishiyama, R. Shah, V.T. Forsyth, E. Mossou et al., Untangling the threads of cellulose mercerization. Nat. Commun. **13**, 6189 (2022). 10.1038/s41467-022-33812-w 36261428 10.1038/s41467-022-33812-w PMC9581993

[35] H.C. Tai, C.H. Chang, W. Cai, J.H. Lin, S.J. Huang et al., Wood cellulose microfibrils have a 24-chain core-shell nanostructure in seed plants. Nat. Plants **9**, 1154–1168 (2023). 10.1038/s41477-023-01430-z 37349550 10.1038/s41477-023-01430-z

[36] X. Shi, Z. Wang, S. Liu, Q. Xia, Y. Liu et al., Scalable production of carboxylated cellulose nanofibres using a green and recyclable solvent. Nat. Sustain. **7**, 315–325 (2024). 10.1038/s41893-024-01267-0

[37] A. Hajian, S.B. Lindström, T. Pettersson, M.M. Hamedi, L. Wågberg, Understanding the dispersive action of nanocellulose for carbon nanomaterials. Nano Lett. **17**, 1439–1447 (2017). 10.1021/acs.nanolett.6b04405 28170274 10.1021/acs.nanolett.6b04405

[38] P.K. Nayak, E.M. Erickson, F. Schipper, T.R. Penki, N. Munichandraiah et al., Review on challenges and recent advances in the electrochemical performance of high capacity Li- and Mn-rich cathode materials for Li-ion batteries. Adv. Energy Mater. **8**, 1702397 (2018). 10.1002/aenm.201702397

[39] S.-L. Cui, M.-Y. Gao, G.-R. Li, X.-P. Gao, Insights into Li-rich Mn-based cathode materials with high capacity: from dimension to lattice to atom. Adv. Energy Mater. **12**, 2003885 (2022). 10.1002/aenm.202003885

[40] R.J. Moon, A. Martini, J. Nairn, J. Simonsen, J. Youngblood, Cellulose nanomaterials review: structure, properties and nanocomposites. Chem. Soc. Rev. **40**, 3941–3994 (2011). 10.1039/C0CS00108B 21566801 10.1039/c0cs00108b

[41] R. Zhang, Z. Hu, Y. Wang, H. Hu, F. Li et al., Single-molecular insights into the breakpoint of cellulose nanofibers assembly during saccharification. Nat. Commun. **14**, 1100 (2023). 10.1038/s41467-023-36856-8 36841862 10.1038/s41467-023-36856-8 PMC9968341

[42] A. Isogai, T. Saito, H. Fukuzumi, TEMPO-oxidized cellulose nanofibers. Nanoscale **3**, 71–85 (2011). 10.1039/c0nr00583e 20957280 10.1039/c0nr00583e

[43] B. Medronho, B. Lindman, Brief overview on cellulose dissolution/regeneration interactions and mechanisms. Adv. Colloid Interface Sci. **222**, 502–508 (2015). 10.1016/j.cis.2014.05.004 24931119 10.1016/j.cis.2014.05.004

[44] J. Cai, L. Zhang, S. Liu, Y. Liu, X. Xu et al., Dynamic self-assembly induced rapid dissolution of cellulose at low temperatures. Macromolecules **41**, 9345–9351 (2008). 10.1021/ma801110g

[45] S. Cichosz, A. Masek, IR study on cellulose with the varied moisture contents: insight into the supramolecular structure. Materials (Basel) **13**, 4573 (2020). 10.3390/ma13204573 33066574 10.3390/ma13204573 PMC7602232

[46] Y. Hishikawa, E. Togawa, T. Kondo, Characterization of individual hydrogen bonds in crystalline regenerated cellulose using resolved polarized FTIR spectra. ACS Omega **2**, 1469–1476 (2017). 10.1021/acsomega.6b00364 31457518 10.1021/acsomega.6b00364 PMC6640953

[47] D. Miyashiro, R. Hamano, K. Umemura, A review of applications using mixed materials of cellulose, nanocellulose and carbon nanotubes. Nanomaterials (Basel) **10**, 186 (2020). 10.3390/nano10020186 31973149 10.3390/nano10020186 PMC7074973

[48] Y.-R. Kang, Y.-L. Li, F. Hou, Y.-Y. Wen, D. Su, Fabrication of electric papers of graphene nanosheet shelled cellulose fibres by dispersion and infiltration as flexible electrodes for energy storage. Nanoscale **4**, 3248–3253 (2012). 10.1039/c2nr30318c 22535335 10.1039/c2nr30318c

[49] A.M. Rao, J. Chen, E. Richter, U. Schlecht, P.C. Eklund et al., Effect of van der Waals interactions on the Raman modes in single walled carbon nanotubes. Phys. Rev. Lett. **86**, 3895–3898 (2001). 10.1103/PhysRevLett.86.3895 11329351 10.1103/PhysRevLett.86.3895

[50] X. Yan, T. Itoh, Y. Kitahama, T. Suzuki, H. Sato et al., A Raman spectroscopy study on single-wall carbon nanotube/polystyrene nanocomposites: mechanical compression transferred from the polymer to single-wall carbon nanotubes. J. Phys. Chem. C **116**, 17897–17903 (2012). 10.1021/jp303509g

[51] D.-S. Ko, J.-H. Park, B.Y. Yu, D. Ahn, K. Kim et al., Degradation of high-nickel-layered oxide cathodes from surface to bulk: a comprehensive structural, chemical, and electrical analysis. Adv. Energy Mater. **10**, 2001035 (2020). 10.1002/aenm.202001035

[52] N. Ogihara, Y. Itou, T. Sasaki, Y. Takeuchi, Impedance spectroscopy characterization of porous electrodes under different electrode thickness using a symmetric cell for high-performance lithium-ion batteries. J. Phys. Chem. C **119**, 4612–4619 (2015). 10.1021/jp512564f

[53] Q. Li, D. Ning, D. Wong, K. An, Y. Tang et al., Improving the oxygen redox reversibility of Li-rich battery cathode materials *via* Coulombic repulsive interactions strategy. Nat. Commun. **13**, 1123 (2022). 10.1038/s41467-022-28793-9 35236854 10.1038/s41467-022-28793-9 PMC8891320

[54] X. Zhu, T.U. Schülli, X. Yang, T. Lin, Y. Hu et al., Epitaxial growth of an atom-thin layer on a LiNi_0.5_Mn_1.5_O_4_ cathode for stable Li-ion battery cycling. Nat. Commun. **13**, 1565 (2022). 10.1038/s41467-022-28963-9 35322022 10.1038/s41467-022-28963-9 PMC8943144

[55] Y. Li, W. Chen, T. Lei, H. Xie, A. Hu et al., Reconstruction suppressed solid-electrolyte interphase by functionalized metal-organic framework. Energy Storage Mater. **59**, 102765 (2023). 10.1016/j.ensm.2023.04.004

[56] Y. Fan, T. Wu, M. He, W. Chen, C. Yan et al., Achieving stable lithium metal anode at 50 mA cm^−2^ current density by LiCl enriched SEI. Small **19**, e2301433 (2023). 10.1002/smll.202301433 37263991 10.1002/smll.202301433

[57] Y. Li, Y. Liu, L. Xue, W. Chen, T. Lei et al., Eliminating anion depletion region and promoting Li^+^ solvation *via* anionphilic metal organic framework for dendrite-free lithium deposition. Nano Energy **92**, 106708 (2022). 10.1016/j.nanoen.2021.106708

[58] C. Wang, L. Xing, J. Vatamanu, Z. Chen, G. Lan et al., Overlooked electrolyte destabilization by manganese (II) in lithium-ion batteries. Nat. Commun. **10**, 3423 (2019). 10.1038/s41467-019-11439-8 31366890 10.1038/s41467-019-11439-8 PMC6668472

[59] J. Betz, J.-P. Brinkmann, R. Nölle, C. Lürenbaum, M. Kolek et al., Cross talk between transition metal cathode and Li metal anode: unraveling its influence on the deposition/dissolution behavior and morphology of lithium. Adv. Energy Mater. **9**, 1900574 (2019). 10.1002/aenm.201900574

[60] A. Hu, W. Chen, F. Li, M. He, D. Chen et al., Nonflammable polyfluorides-anchored quasi-solid electrolytes for ultra-safe anode-free lithium pouch cells without thermal runaway. Adv. Mater. **35**, e2304762 (2023). 10.1002/adma.202304762 37669852 10.1002/adma.202304762

[61] Y.-G. Cho, S.-H. Jung, S.H. Joo, Y. Jeon, M. Kim et al., A metal-ion-chelating organogel electrolyte for Le Chatelier depression of Mn^3+^ disproportionation of lithium manganese oxide spinel. J. Mater. Chem. A **6**, 22483–22488 (2018). 10.1039/C8TA08560A

[62] J. Mun, J.-H. Park, W. Choi, A. Benayad, J.-H. Park et al., New dry carbon nanotube coating of over-lithiated layered oxide cathode for lithium ion batteries. J. Mater. Chem. A **2**, 19670–19677 (2014). 10.1039/C4TA04818K

[63] H. Yang, Y. Wan, K. Sun, M. Zhang, C. Wang et al., Reconciling mass loading and gravimetric performance of MnO_2_ cathodes by 3D-printed carbon structures for zinc-ion batteries. Adv. Funct. Mater. **33**, 2215076 (2023). 10.1002/adfm.202215076

[64] J.-M. Kim, J.A. Kim, S.-H. Kim, I.S. Uhm, S.J. Kang et al., All-nanomat lithium-ion batteries: a new cell architecture platform for ultrahigh energy density and mechanical flexibility. Adv. Energy Mater. **7**, 1701099 (2017). 10.1002/aenm.201701099

[65] J.-H. Kim, Y.-H. Lee, S.-J. Cho, J.-G. Gwon, H.-J. Cho et al., Nanomat Li–S batteries based on all-fibrous cathode/separator assemblies and reinforced Li metal anodes: towards ultrahigh energy density and flexibility. Energy Environ. Sci. **12**, 177–186 (2019). 10.1039/C8EE01879K

[66] S.-H. Kim, N.-Y. Kim, U.-J. Choe, J.-M. Kim, Y.-G. Lee et al., Ultrahigh-energy-density flexible lithium-metal full cells based on conductive fibrous skeletons. Adv. Energy Mater. **11**, 2100531 (2021). 10.1002/aenm.202100531

[67] J.-M. Kim, C.-H. Park, Q. Wu, S.-Y. Lee, Cathodes: 1D building blocks-intermingled heteronanomats as a platform architecture for high-performance ultrahigh-capacity lithium-ion battery cathodes. Adv. Energy Mater. **6**, 1670008 (2016). 10.1002/aenm.201670008

[68] Y. Jiao, S. Wang, Y. Ma, M. Zhou, L. Zhang et al., Tailoring interfacial derivative for lithium–sulfur pouch cells with ultra-long cycling performance. Adv. Energy Mater. **13**, 2301233 (2023). 10.1002/aenm.202301233

