# Supplementary material for: Cellulose Elementary Fibrils as Deagglomerated Binder for High-Mass-Loading Lithium Battery Electrodes
Source: Nanomicro Lett. 2025 Jan 21;17:112. doi: 10.1007/s40820-024-01642-8 (PMC11751347; doi:10.1007/s40820-024-01642-8)
Supplement: Supplementary file 1 — Supplementary file1 (DOCX 2896 KB) [file 40820_2024_1642_MOESM1_ESM.docx]

Supporting Information for

**Cellulose Elementary Fibrils as Deagglomerated Binder for High-Mass-Loading Lithium Battery Electrodes**

Young-Kuk Hong^1^, Jung-Hui Kim^1^, Nag-Young Kim^1^, Kyeong-Seok Oh^1^, Hong-I Kim^1^, Seokhyeon Ryu^1^, Yumi Ko^1^, Ji-Young Kim^2^, Kwon-Hyung Lee^3^, and Sang-Young Lee^1,4^*

^1^ Department of Chemical and Biomolecular Engineering, Yonsei University, 50 Yonsei-ro, Seodaemun-gu, Seoul, 03722, Republic of Korea

^2^ Advanced Analysis & Data Center, Korea Institute of Science and Technology (KIST), Seoul 02792, Republic of Korea

^3^ Ulsan Advanced Energy Technology R&D Center, Korea Institute of Energy Research (KIER), Ulsan, 44776, Republic of Korea

^4^ Department of Battery Engineering, Yonsei University, 50 Yonsei-ro, Seodaemun-gu, Seoul, 03722, Republic of Korea

*Corresponding author. E-mail: [syleek@yonsei.ac.kr](mailto:syleek@yonsei.ac.kr) (Sang-Young Lee)

**Supplementary Figures and Tables**

**
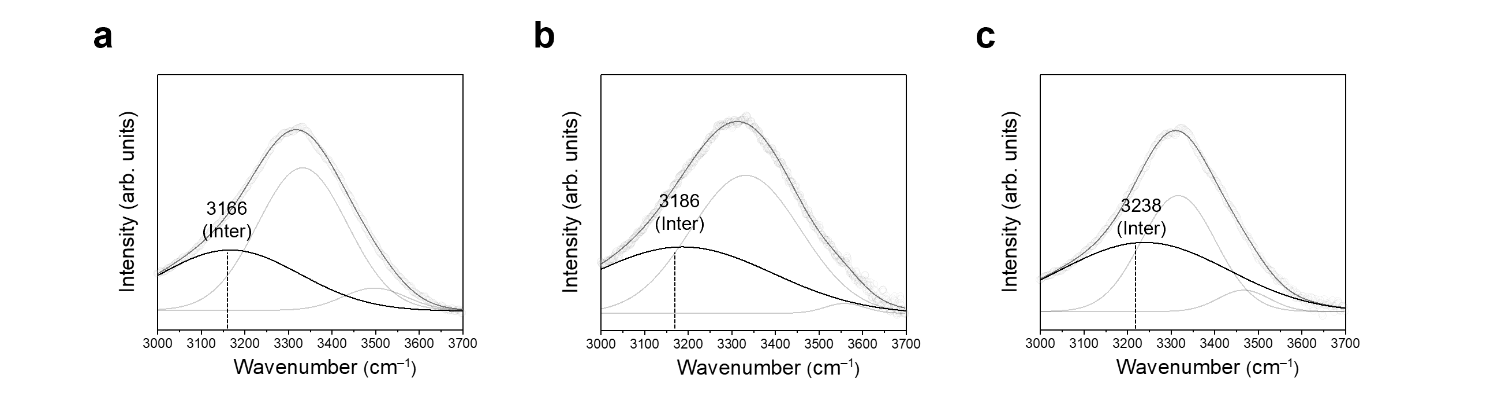
**

**Fig. S1** FT-IR spectras of the cellulose in aqueous suspensions. **a** CNF(-OH), **b** CNF(-COO^–^), and **c** CEF(-COO^–^)


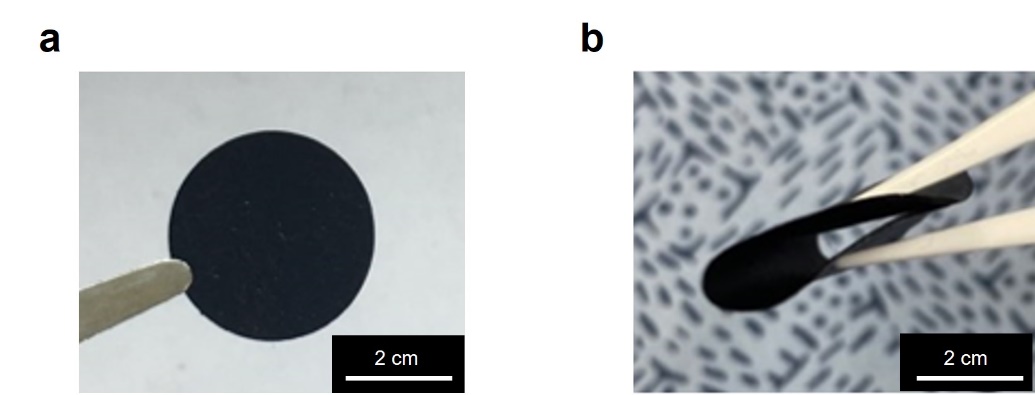


**Fig. S2** Photographs of **a** OLO cathode with CEF binder and **b** upon bending deformation

**
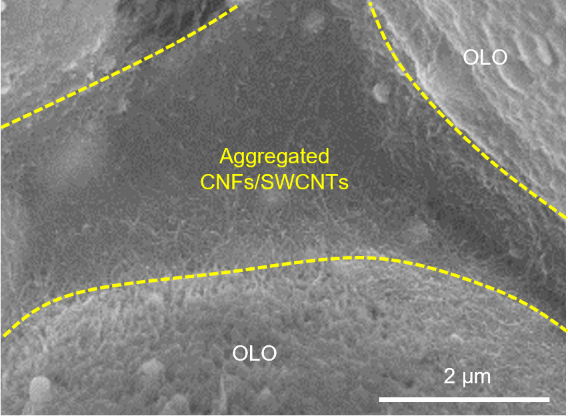
**

**Fig. S3** SEM image of the CNF(-COO^–^) cathode

**
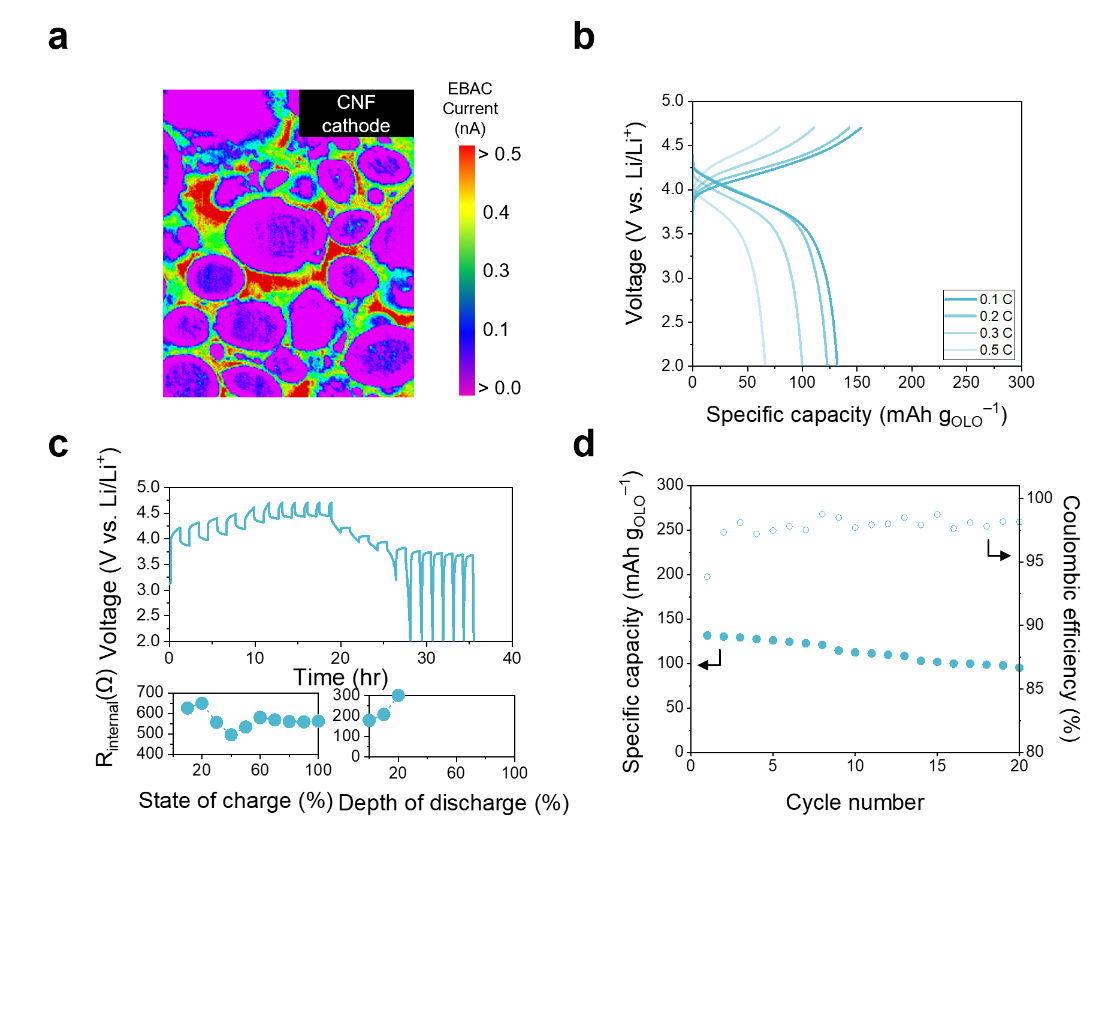
**

**F****i****g. S4** Electrochemical performance of cells (CNF(-COO^–^) cathode (areal-mass-loading = 19 mg cm^–2^)||Li metal anode (100 μm)). **a** Spatial distribution of electronic resistance of the CNF cathode using EBAC analysis. **b** Charge/discharge voltage profiles at varied discharge current rates (0.1 C (=0.47 mA cm^–2^) – 0.5 C (=2.3 mA cm^–2^)) at a fixed charge current rate of 0.1 C. **c** (top) GITT profile upon repeated current stimuli at charge/discharge current rate of 0.1 C/0.1 C (=0.47 mA cm^–2^) and (bottom) internal cell resistance (*R*_internal_) as a function of SOC and DOD. **d** Cycling retention at charge/discharge current rates of 0.2 C/0.2 C (=0.95 mA cm^–2^/0.95 mA cm^–2^) under a voltage range of 2.0–4.7 V


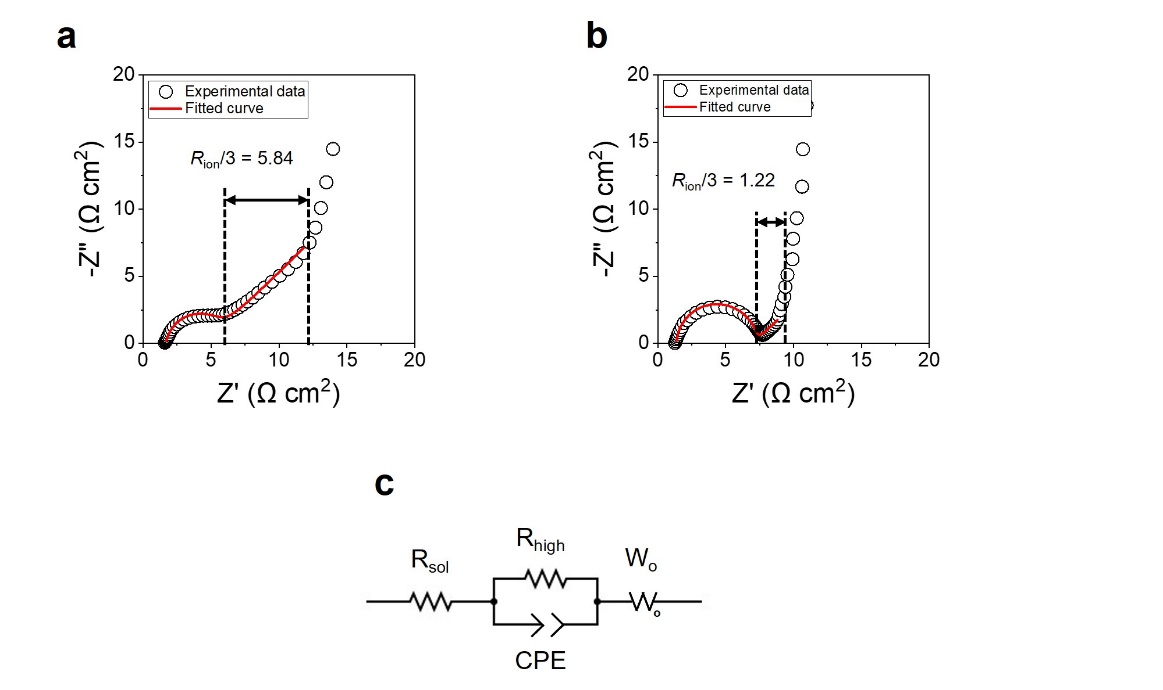


**Fig****. S5** Nyquist plots of **a** PVDF and **b** CEF cathodes obtained by symmetric cells configuration at 0 % SOC, in which symbols and solid lines represent experimental data and fitted curves based on a transmission line equivalent circuit model (TLM), respectively. **c** Equivalent circuits utilizing generalized finite length Warburg element open circuit terminus (W_o_)

**
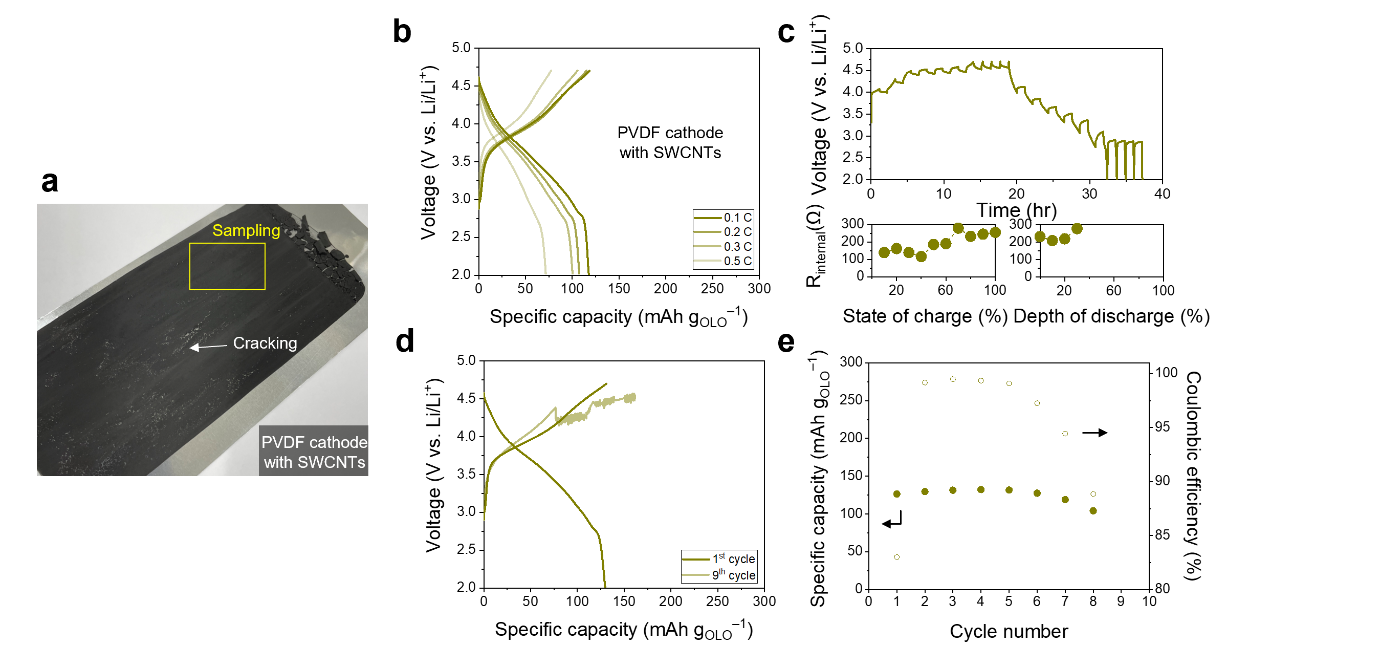
**

**Fig. S6** Electrochemical performance of cells (PVDF cathode with SWCNTs (areal-mass-loading = 19 mg cm^–2^)||Li metal anode (100 μm)). **a** Photograph. **b** Charge/discharge voltage profiles at varied discharge current rates (0.1 C (=0.47 mA cm^–2^) – 0.5 C (=2.3 mA cm^–2^)) at a fixed charge current rate of 0.1 C. **c** (top) GITT profile upon repeated current stimuli at charge/discharge current rate of 0.1 C/0.1 C (=0.47 mA cm^–2^) and (bottom) internal cell resistance (*R*_internal_) as a function of SOC and DOD. **d** Charge/discharge voltage profiles at 1^st^ and 9^th^ cycles. **e** Cycling performance at charge/discharge current rates of 0.2 C/0.2 C (=0.95 mA cm^–2^/0.95 mA cm^–2^) under a voltage range of 2.0–4.7 V

*
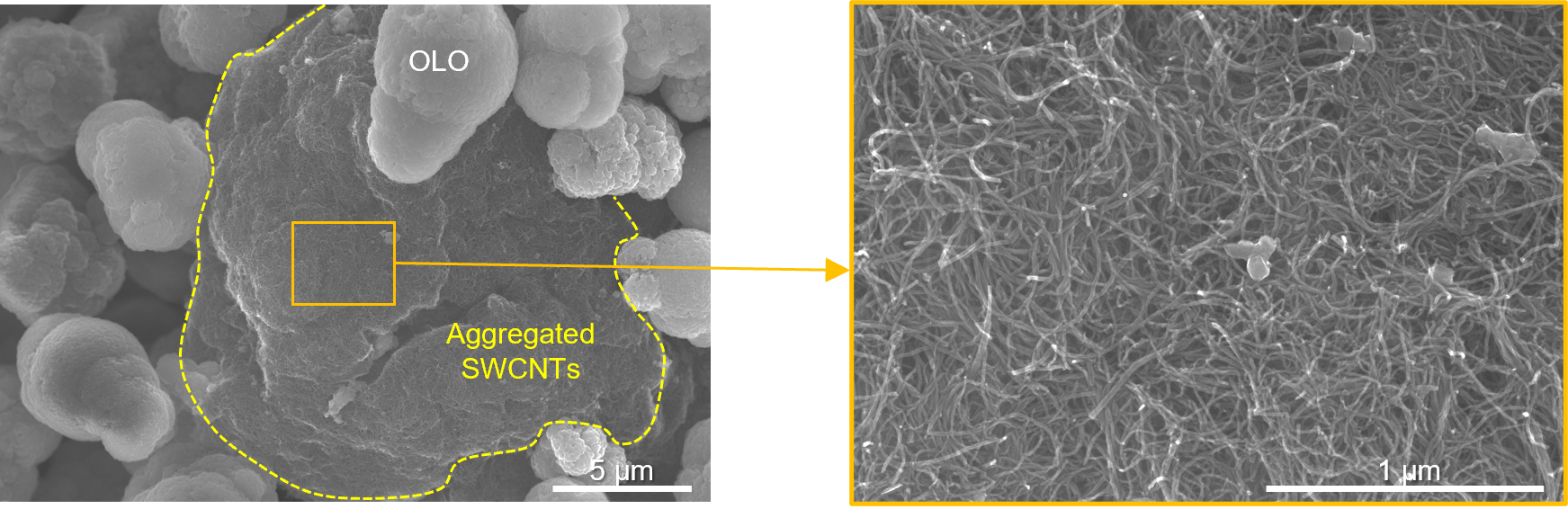
*

**Fig. S7** SEM images of the PVDF cathode containing the SWCNTs

**
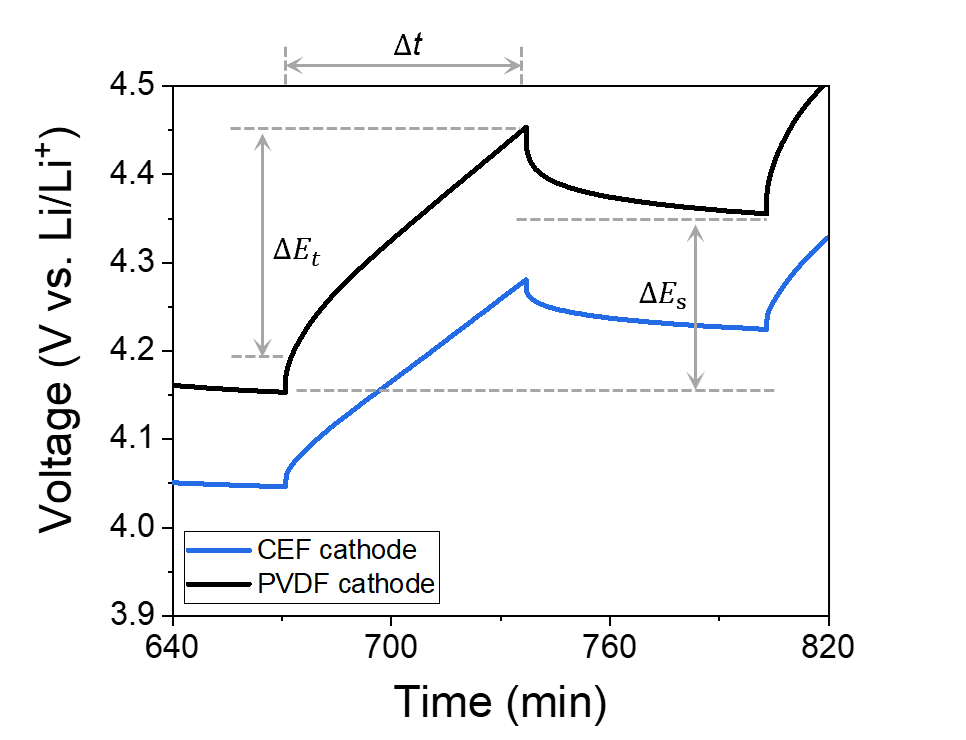
**

**Fig. S8** Galvanostatic intermittent titration technique (GITT) profiles of the CEF and PVDF (areal-mass-loading = 22 mg cm^–2^) cathodes at a current rate of 0.1 C (= 0.55 mA cm^–2^) with the interruption time of 1 h between the pulses. The Li^+^ diffusion coefficients ($D_{Li+})$were calculated using the following equation (S1) [1]:

$D_{Li+}$= $\frac{4}{\pi\Delta t}\left( \frac{m_{B}V_{M}}{M_{B}S} \right)^{2}\left( \frac{{\Delta E}_{s}}{{\Delta E}_{t}} \right)^{2}$ (S1)

where $m_{B}$ is assigned to the mass of the electrode active material, $S$ is the geometric area of the electrode, $M_{B}$ is the molar mass of the electrode material, $V_{M}$ is the molar volume of the electrode material, and other parameters ($\Delta t$, ${\Delta E}_{t}$, and ${\Delta E}_{s}$) in the equation are displayed in the GITT profiles shown above

|  | Electrolyte composition |
| --- | --- |
| Electrolyte 1 | 1 M LiPF_6_ |
| Electrolyte 2 | 0.3 M Mn(TFSI)_2_ |
| Electrolyte 3 | 1 M LiPF_6_ + 0.3 M Mn(TFSI)_2_ |
| Electrolyte 4 | 1 M LiPF_6_ + 0.3 M Mn(TFSI)_2_ with CEF |


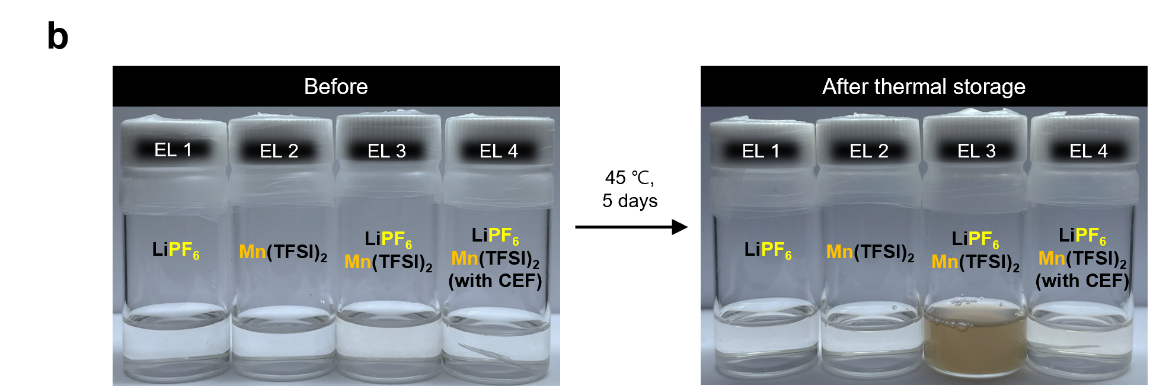

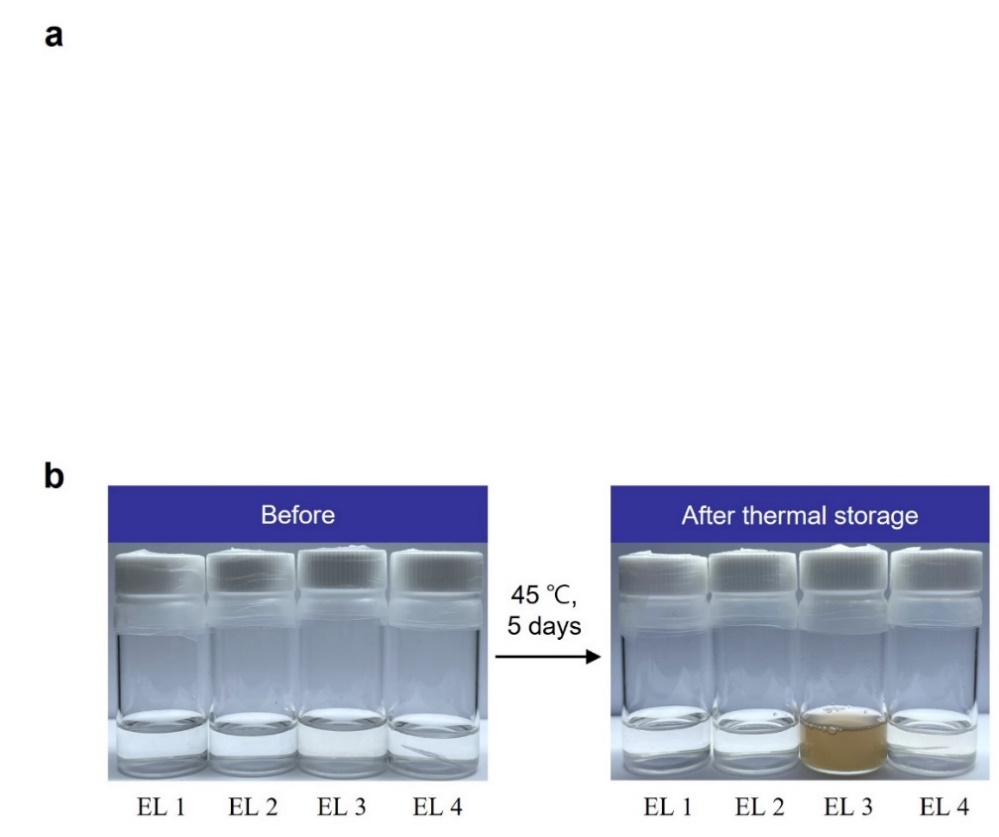


**Fig. S9** Electrolyte degradation test under coexistence of PF_6_^–^ and Mn^2+^. **a** Compositions of the carbonate-based liquid electrolytes. **b** Photographs of the carbonate-based liquid electrolytes before (left) and after (right) being stored for 5 days at 45 ℃, using a solvent mixture of EC/DMC (1/1, v/v) in the whole samples

**
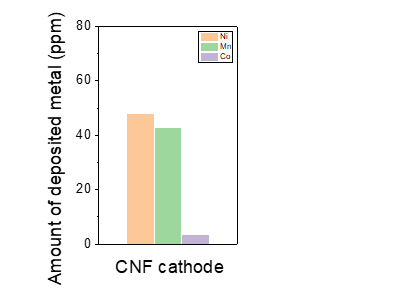
**

**F****ig. S10** Amount of metallic Ni, Mn and Co deposited on cycled Li metal anodes paired with the CNF cathode, measured by ICP-MS analysis


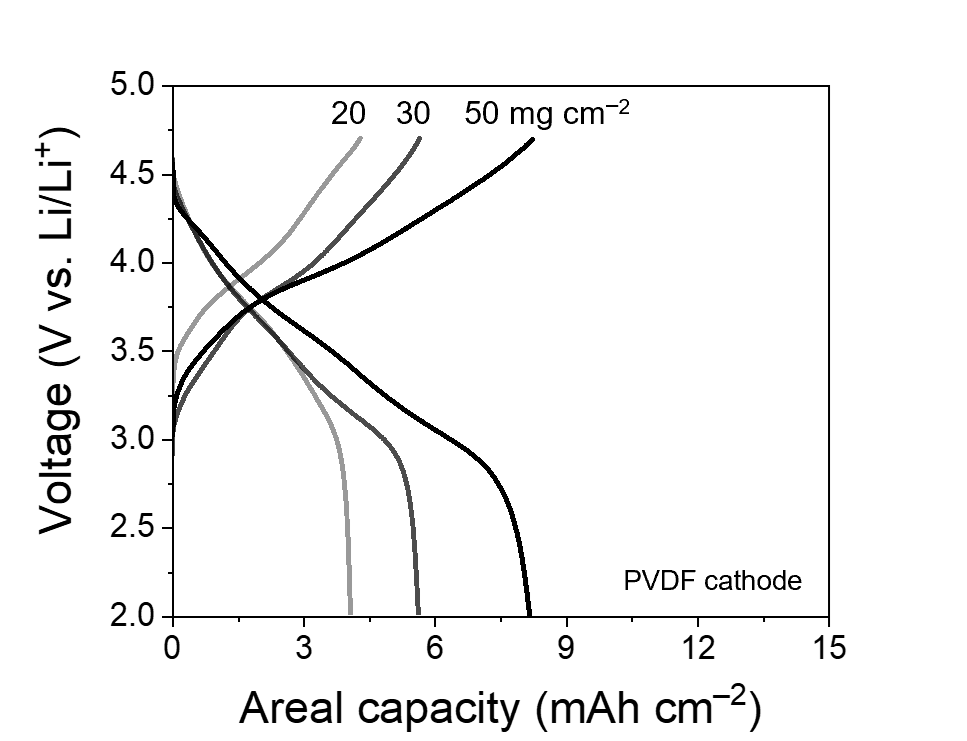


**Fig. S11** Charge/discharge voltage profiles of the cells as a function of areal-mass-loading of the PVDF cathodes at charge/discharge current rates of 0.05C/0.1C and voltage range of 2.0–4.7 V

**
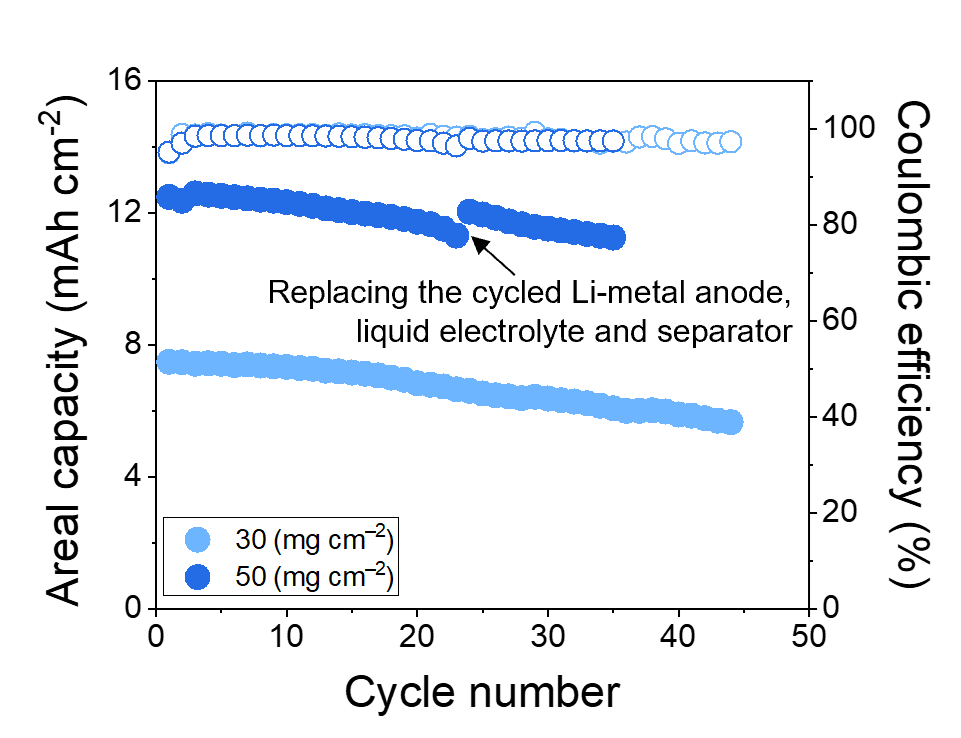
**

**Fi****g. S12** Cycling performance of cells (CEF cathode (areal-mass-loading = 30 and 50 mg cm^–2^)||Li metal anode (100 μm)) at charge/discharge current rates of 0.2C/0.2C and voltage range of 2.0–4.7 V

We evaluated the cycling performance of CEF cathodes with high areal-mass-loadings of 30 and 50 mg cm^−2^. Both cathodes showed stable capacity retention during cycling. However, the CEF cathode with an areal-mass-loading of 50 mg cm^−2^ showed relatively limited cycle life (~23 cycles). When conventional carbonate electrolytes are used, Li metal anodes tend to show severely low plating/stripping reversibility, which becomes more pronounced at higher plating/stripping capacities [S2]. In this study, approximately 35 μm-thick Li (paired with the 30 mg cm⁻² cathode) and 59 μm-thick Li (paired with the 50 mg cm⁻² cathode) were plated and stripped from the Li metal anode during each cycle, which initially had a thickness of 100 μm. To identify the cause of the capacity fading, we replaced the cycled Li metal anode, separator, and electrolyte with fresh components after observing the capacity fading. The cell then recovered close to its initial discharge capacity, indicating that the CEF cathode may not be the primary factor contributing to cycling decay. Similar experiments involving the replacement of the cycled Li metal anode, separator, and liquid electrolyte have been reported in previous studies for Li-metal cells [S3, S4].


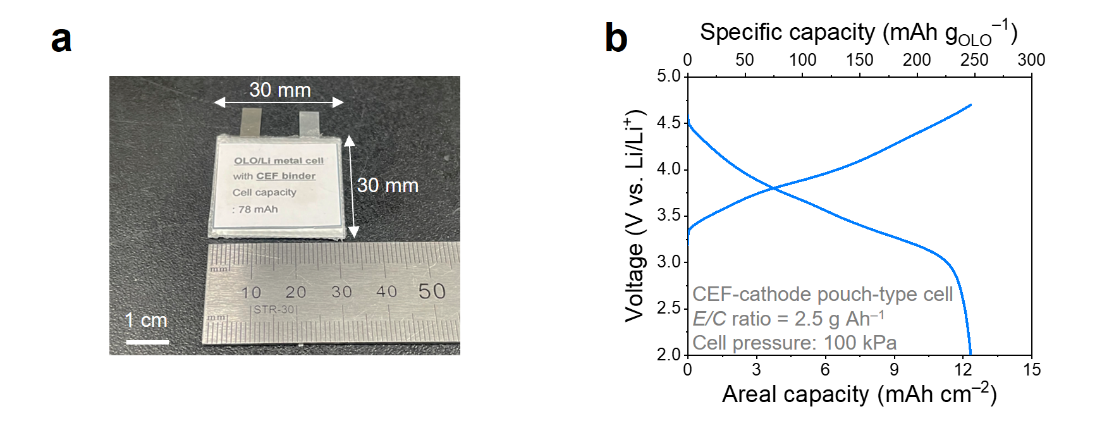
**Fig.** **S13** (**a**) Photograph of the pouch-type cell (30 × 30 mm^2^) containing CEF cathode (areal-mass-loading of 50 mg cm^–2^). (**b**) Charge/discharge voltage profile at charge/discharge current rates of 0.05C/0.1C and voltage range of 2.0–4.7 VThe $D_{Li+}$ was calculated using the following equation (S2) [S1]:

$D_{Li+}$= $\frac{4}{\pi\Delta t}\left( \frac{m_{B}V_{M}}{M_{B}S} \right)^{2}\left( \frac{{\Delta E}_{s}}{{\Delta E}_{t}} \right)^{2}$ (S2)

where $m_{B}$ is assigned to the mass of the electrode active material, $S$ is the geometric area of the electrode, $M_{B}$ is the molar mass of the electrode material, $V_{M}$ is the molar volume of the electrode material, and other parameters ($\Delta t$, ${\Delta E}_{t}$, and ${\Delta E}_{s}$) in the equation were displayed in the Fig. S5.

**Table S1** Calculation details for the $D_{Li+}$ of the CEF and PVDF cathodes

|  | $\Delta t$ | $M_{B}$ | $V_{M}$ | $m_{B} S^{-1}$ | ${\Delta E}_{s}$ | ${\Delta E}_{t}$ | $D_{Li+}$ |
| --- | --- | --- | --- | --- | --- | --- | --- |
|  | (s) | (g mol^–1^) | (cm^3^ mol^–1^) | (mg cm^–2^) | (mV) | (mV) | (cm^2^ s^–1^) |
| CEF cathode | 3600 | 106.32 | 20.53 | 22.00 | 178.23 | 218.9 | 4.23 x 10^-9^ |
| PVDF  cathode | 3600 | 106.32 | 20.53 | 22.00 | 202.24 | 281.52 | 3.29 x 10^-9^ |

As illustrated in Fig. 6e, the specific energies of the full cells is plotted. The equation be derived according to,

Specific energy (Wh kg^–1^) = $\frac{\mathrm{Energy}}{Mass of cell}$ = $\frac{Norminal voltage \times Cell capacity}{M_{\mathrm{cathode}} + M_{\mathrm{anode}} + M_{\mathrm{separator}} + M_{\mathrm{electrolyte}}}$

where *M*_cathode_, *M*_anode_, and *M*_electrolyte_ are the mass of cathode, anode (comprising Li metal (100 μm corresponding to an areal capacity of 20 mAh cm^–2^) and Cu current collector (9 μm)), separator and injected liquid electrolyte. *C* and *A* indicates capacity and area, respectively. The electrolyte mass/cell capacity (*E*/*C*) ratio in the cell was controlled as 2.5 g Ah^–1^. The nominal voltage of the cell was set as 3.6 V.

**Table S2** Calculation details for the specific energy densities of the cells containing the CEF-based OLO cathodes

| *C*/*A* | *M*_cathode_/A | *M*_anode_/A | *M*_separator_/A | *M*_electrolyte_/A | *M*_total_/A | Specific energy |
| --- | --- | --- | --- | --- | --- | --- |
| (mAh cm^–2^) | (mg cm^–2^) | (mg cm^–2^) | (mg cm^–2^) | (mg cm^–2^) | (mg cm^–2^) | (Wh kg^–1^) |
| 12.5 | 55.6 | 13.2 | 0.98 | 31.25 | 101.03 | 445.4 |

**Table S3** Comparison of the CEF-based OLO full cells (this study) with previously reported OLO cathodes. Note that some previous works did not reveal the thickness data of Li-metal anodes. Therefore, these values were assumed to be 100 μm

|  | C/A | *M*_cathode_/A | *M*_anode_/A | *M*_separator_/A | *M*_electrolyte_/A | *M*_total_/A | Specific energy |
| --- | --- | --- | --- | --- | --- | --- | --- |
|  | (mAh cm^–2^) | (mg cm^–2^) | (mg cm^–2^) | (mg cm^–2^) | (mg cm^–2^) | (mg cm^–2^) | (Wh kg^–1^) |
| CEF cathode | 12.5 | 55.64 | 13.2 | 0.98 | 31.25 | 101.03 | 445.4 |
| PVDF cathode | 9.15 | 60.96 | 13.2 | 0.98 | 31.25 | 106.39 | 309.6 |
| Hybrid binder [S5] | 0.81 | 8.71 | 13.2 | 0.98 | 1.88 | 24.77 | 117.7 |
| Guar gum  [S6] | 0.38 | 7.90 | 13.2 | 0.98 | 1.25 | 23.33 | 58.6 |
| Polymeric nanofibers  [S7] | 1.60 | 10.00 | 13.2 | 0.98 | 4.06 | 28.24 | 203.9 |
| Sodium-alginate  [S8] | 0.50 | 7.92 | 13.2 | 0.98 | 1.25 | 23.35 | 77.1 |
| Fluorinated polyimide  [S9] | 0.69 | 9.40 | 13.2 | 0.98 | 1.88 | 25.46 | 97.6 |
| Aqueous binder  [S10] | 0.58 | 8.11 | 13.2 | 0.98 | 1.44 | 23.72 | 87.9 |
| Double-helix-superstructure  [S11] | 1.25 | 11.65 | 13.2 | 0.98 | 3.13 | 28.96 | 155.4 |
| Water-soluble  guar gum  [S12] | 0.31 | 7.02 | 13.2 | 0.98 | 0.81 | 22.01 | 50.7 |
| DNA-wrapped  CNTs [S13] | 1.80 | 14.10 | 13.2 | 0.98 | 5.00 | 33.28 | 194.7 |
| Polyacrylic  acid [S14] | 0.55 | 7.91 | 13.2 | 0.98 | 1.25 | 23.34 | 84.8 |
| Nanofibrous  carbon binder [S15]^]^ | 1.61 | 13.00 | 13.2 | 0.98 | 4.38 | 31.56 | 183.7 |
| Carbon fibrous skeletons  [S16] | 2.85 | 16.00 | 13.2 | 0.98 | 6.50 | 36.68 | 279.7 |

**Supplementary References**

1. W. Weppner, R. A. Huggins, Determination of the kinetic parameters of mixed‐conducting electrodes and application to the system Li_3_Sb. J. Electrochem. Soc. **124**, 1569 (1977). <https://doi.org/10.1149/1.2133112>
2. H. Wang, Z. Yu, X. Kong, S. C. Kim, D. T. Boyle et al., Liquid electrolyte: The nexus of practical lithium metal batteries. Joule **6**, 588-616 (2022). <https://doi.org/10.1016/j.joule.2021.12.018>
3. L. Qie, C. Zu, A. Manthiram, A high energy lithium-sulfur battery with ultrahigh-loading lithium polysulfide cathode and its failure mechanism. Adv. Energy Mater. **6**, 1502459 (2016). <https://doi.org/10.1002/aenm.201502459>
4. J.-H. Kim, J.-M. Kim, S.-K. Cho, N.-Y. Kim, S.-Y. Lee, Redox-homogeneous, gel electrolyte-embedded high-mass-loading cathodes for high-energy lithium metal batteries. Nat. Commun. **13**, 2541 (2022). <https://doi.org/10.1038/s41467-022-30112-1>
5. K. Notake, T. Gunji, H. Kokubun, S. Kosemura, Y. Mochizuki et al., The application of a water-based hybrid polymer binder to a high-voltage and high-capacity Li-rich solid-solution cathode and its performance in Li-ion batteries. J. Appl. Electrochem. **46**, 267-278 (2016). <https://doi.org/10.1007/s10800-016-0930-8>
6. T. Zhang, J.-t. Li, J. Liu, Y.-p. Deng, Z.-g. Wu et al., Suppressing the voltage-fading of layered lithium-rich cathode materials via an aqueous binder for Li-ion batteries. Chem. Commun. **52**, 4683-4686 (2016). <https://doi.org/10.1039/c5cc10534j>
7. J. M. Kim, J. A. Kim, S. H. Kim, I. S. Uhm, S. J. Kang et al., All‐nanomat lithium‐ion batteries: A new cell architecture platform for ultrahigh energy density and mechanical flexibility. Adv. Energy Mater. **7**, 1701099 (2017). <https://doi.org/10.1002/aenm.201701099>
8. S. J. Zhang, Y. P. Deng, Q. H. Wu, Y. Zhou, J. T. Li et al., Sodium‐alginate‐based binders for lithium‐rich cathode materials in lithium‐ion batteries to suppress voltage and capacity fading. ChemElectroChem **5**, 1321-1329 (2018). <https://doi.org/10.1002/celc.201701358>
9. H. Q. Pham, G. Kim, H. M. Jung, S. W. Song, Fluorinated polyimide as a novel high‐voltage binder for high‐capacity cathode of lithium‐ion batteries. Adv. Funct. Mater. **28**, 1704690 (2018). <https://doi.org/10.1002/adfm.201704690>
10. Kazzazi, D. Bresser, A. Birrozzi, J. von Zamory, M. Hekmatfar et al., Comparative analysis of aqueous binders for high-energy Li-rich NMC as a lithium-ion cathode and the impact of adding phosphoric acid. ACS Appl. Mater. Interfaces **10**, 17214-17222 (2018). <https://doi.org/10.1021/acsami.8b03657>
11. G. Zhang, B. Qiu, Y. Xia, X. Wang, Q. Gu et al., Double-helix-superstructure aqueous binder to boost excellent electrochemical performance in Li-rich layered oxide cathode. J. Power Sources **420**, 29-37 (2019). <https://doi.org/10.1016/j.jpowsour.2019.02.086>
12. Z.-W. Yin, T. Zhang, S.-J. Zhang, Y.-P. Deng, X.-X. Peng et al., Understanding the role of water-soluble guar gum binder in reducing capacity fading and voltage decay of Li-rich cathode for Li-ion batteries. Electrochim. Acta **351**, 136401 (2020).
    <https://doi.org/10.1016/j.electacta.2020.136401>
13. J. M. Kim, J. H. Park, E. Jo, H. S. Kim, S. H. Kim et al., Ecofriendly chemical activation of overlithiated layered oxides by DNA‐wrapped carbon nanotubes. Adv. Energy Mater. **10**, 1903658 (2020). <https://doi.org/10.1002/aenm.201903658>
14. J. Yang, P. Li, F. Zhong, X. Feng, W. Chen et al., Suppressing voltage fading of Li‐rich oxide cathode via building a well‐protected and partially‐protonated surface by polyacrylic acid binder for cycle‐stable Li‐ion batteries. Adv. Energy Mater. **10**, 1904264 (2020). <https://doi.org/10.1002/aenm.201904264>
15. J.-M. Kim, S.-H. Kim, N. Y. Kim, M.-H. Ryou, H. Bae et al., Nanofibrous conductive binders based on DNA-wrapped carbon nanotubes for lithium battery electrodes. Iscience. **23**, 101739 (2020). <https://doi.org/10.1016/j.isci.2020.101739>
16. S. H. Kim, N. Y. Kim, U. J. Choe, J. M. Kim, Y. G. Lee et al., Ultrahigh‐energy‐density flexible lithium‐metal full cells based on conductive fibrous skeletons. Adv. Energy Mater. **11**, 2100531 (2021). <https://doi.org/10.1002/aenm.202100531>
